# The effect of *G0S2* on insulin sensitivity: A proteomic analysis in a *G0S2*-overexpressed high-fat diet mouse model

**DOI:** 10.3389/fendo.2023.1130350

**Published:** 2023-03-23

**Authors:** Dongming Wu, Zhenyuan Zhang, Wenxiu Sun, Yong Yan, Mengzhe Jing, Shizhan Ma

**Affiliations:** ^1^ College of First Clinical Medicine, Shandong University of Traditional Chinese Medicine, Jinan, China; ^2^ Department of Endocrinology, Shandong Provincial Hospital Affiliated to Shandong First Medical University, Jinan, China; ^3^ Shandong Clinical Research Center of Diabetes and Metabolic Diseases, Jinan, China; ^4^ Shandong Key Laboratory of Endocrinology and Lipid Metabolism, Jinan, China; ^5^ Shandong Prevention and Control Engineering Laboratory of Endocrine and Metabolic Diseases, Jinan, China; ^6^ Department of Nursing, Taishan Vocational College of Nursing, Taian, China; ^7^ Department of Transfusion Medicine, Shandong Provincial Hospital Affiliated to Shandong First Medical University, Jinan, China

**Keywords:** *G0S2*, insulin sensitivity, label-free proteomics, metabolic diseases, high-fat diet

## Abstract

**Background:**

Previous research has shown a tight relationship between the G0/G1 switch gene 2 (G0S2) and metabolic diseases such as non-alcoholic fatty liver disease (NAFLD) and obesity and diabetes, and insulin resistance has been shown as the major risk factor for both NAFLD and T2DM. However, the mechanisms underlying the relationship between G0S2 and insulin resistance remain incompletely understood. Our study aimed to confirm the effect of G0S2 on insulin resistance, and determine whether the insulin resistance in mice fed a high-fat diet (HFD) results from G0S2 elevation.

**Methods:**

In this study, we extracted livers from mice that consumed HFD and received tail vein injections of AD-G0S2/Ad-LacZ, and performed a proteomics analysis.

**Results:**

Proteomic analysis revealed that there was a total of 125 differentially expressed proteins (DEPs) (56 increased and 69 decreased proteins) among the identified 3583 proteins. Functional enrichment analysis revealed that four insulin signaling pathway-associated proteins were significantly upregulated and five insulin signaling pathway -associated proteins were significantly downregulated.

**Conclusion:**

These findings show that the DEPs, which were associated with insulin resistance, are generally consistent with enhanced insulin resistance in G0S2 overexpression mice. Collectively, this study demonstrates that G0S2 may be a potential target gene for the treatment of obesity, NAFLD, and diabetes.

## Introduction

The increasing incidence of metabolic diseases such as obesity, type 2 diabetes mellitus (T2DM), dyslipidemia, and nonalcoholic fatty liver disease (NAFLD) that are triggered by metabolic derangements has been a subject of serious concern worldwide in the past few decades. The systemic metabolic dyshomeostasis caused by impaired insulin signaling is a hallmark of metabolic disease (1, 2). The overabundance of circulating fatty acids lead to insulin resistance, and the aggravation of insulin resistance can further inhibit the antilipolytic effect of insulin and increase lipolysis. The decrease in fatty acid oxidation and increase in cytosolic levels of free fatty acids increases the overall risk of T2DM. The accumulation of lipids in the liver leads to hepatic insulin resistance and NAFLD Therefore, insulin resistance is the most important etiological factor of metabolic disorders (3, 4). Accumulating evidence has shown that simultaneous presence of obesity, NAFLD, and type 2 diabetes mellitus (T2DM) is frequently observed and acts synergistically, resulting in an increased risk of hepatic and cardiovascular clinical outcomes (5–7).

The G0/G1 switch gene 2 (*G0S2*), also known as the lipolytic inhibitor, was originally identified in lymphocytes during the phase of G0 to G1 cell cycle transition that is associated with pharmaceutical stimulation (8, 9). *G0S2* encodes a small 12-kDa protein and is abundantly expressed in the liver, adipose tissue, heart, and skeletal muscle (10, 11). In humans and mice, G0S2 is a multifaceted protein and has been shown to play various important roles in metabolism (9, 10). G0S2 mediates endoplasmic reticulum stress-induced metabolism dysfunction in mice models with metabolic disorders through the PERK-eIF2α-ATF4 pathway (12). As the rate limiting step in fat catabolism, *G0S2* knockout mice shows enhanced lipid metabolism, enhanced thermogenesis, and improved insulin sensitivity (13).

The liver is one of the primary metabolic organs involved in energy homeostasis and glycolipid metabolism and disposes off as much as one-third of the glucose and lipid load (14). Insulin resistance is a primary characteristic and underlying cause of metabolic disorders, including non-alcoholic fatty liver disease (NAFLD) (15). Liver insulin resistance in NAFLD increase the risk for metabolic diseases such as T2DM (16, 17). It has been shown that insulin resistance in adipose tissue contributes to excessive release of fatty acids into the bloodstream, which are taken up by the liver, resulting in liver insulin resistance and NAFLD through dysregulated lipolysis (18–20). It has been revealed that loss of liver glycogen synthesis, which promotes and diverts glucose toward fat synthesis, is the result of liver insulin resistance. G0S2 plays an important role in inducing hepatic steatosis through downregulation of UPR signaling, while regulating lipolysis and energy metabolism by inhibiting adipose triglyceride lipase (ATGL) (14, 21). G0S2 has been shown to exert significant influence on the metabolism of liver lipids, while it has been shown that lipid metabolism has a close relationship with insulin sensitivity (15–17). G0S2 expression was upregulated in the hepatocytes of Nagoya-Shibata-Yasuda (NSY) mice fed with high-sucrose diet (22). G0S2 can modulate the lipolysis process by interacting with ATGL, and the level of G0S2 is upregulated in the occurrence of fatty liver disease in mice (9, 14). Thus far, the precise underlying mechanisms of G0S2 in the regulation of insulin resistance-related NAFLD are still unknown. To reveal the mechanism of G0S2 in NAFLD, we performed a preliminary study of proteomic analysis of livers taken from G0S2-overexpressed mice fed high-fat diet (HFD) and control mice fed HFD by using quantitative proteomics, GO analysis, and KEGG analysis. This study shows that overexpression of the *G0S2* gene aggravates liver insulin resistance of mice through upregulating P-Foxo1, Socs3, and Ptpn1 and downregulating Gstp1 and Ppar-γ, which demonstrates that G0S2 may be a potential target gene for the treatment of NAFLD, obesity, and diabetes.

## Materials and methods

### Animal models

Eight-week-old male C57BL/6 mice were used in this study. The mice were housed in microisolator cages in a specific pathogen-free (SPF) animal room maintained at a controlled environment of temperature of 22 ± 2°C and humidity of 55%, under a 12-h light/dark cycle. Mice had *ad libitum* access to water and high-fat diet (HFD) (protein, 20 kcal%; fat, 45 cal%; carbohydrates, 35 kcal%, D12451, Research Diets, New Brunswick, NJ, USA) for 12 weeks. We selected the mice in *G0S2* overexpression group to receive tail vein injections of Ad-*G0S2* (2.51×10^10^ PFU/mL), and the control mice were injected with Ad-LacZ (4.5×10^10^ PFU/mL) *via* the tail vein as control. Following the operation, all mice continued on the existing diet for 4 weeks. Body weight and glucose tolerance levels were monitored routinely. At the end point, mice were euthanized to minimize suffering, and the livers were extracted, frozen, and stored in liquid nitrogen. All animal experiments in this protocol were approved by The Animal Care and Use Committee of Shandong Provincial Hospital.

Body weight was measured at the same time every week during the experiments. For the glucose tolerance test (GTT) and insulin tolerance test (ITT), mice were fasted for 6 h, and blood glucose was measured after intraperitoneal injection of glucose (2 g/kg body weight) and insulin (0.75 U/kg body weight), respectively. Blood glucose levels were measured at 15, 30, 60, 90, and 120 minutes after the glucose or insulin injection.

### Tissue sample preparation

To the lysis samples, the SDT buffer (4% SDS, 100 mM Tris-HCl, 1 mM DTT, pH 7.6) was added to the liver tissues, and an Automated Homogenizer (MP Fastprep-24, 6.0M/S, 30S) was used to homogenize the lysate twice. Boiling, centrifugation, and filtration were used to extract the homogenate supernatant. The amount of protein was quantified as previously described (23). The protein extracts were digested with trypsin based on a filter-aided sample preparation (FASP) procedure (24). Next, 12.5% SDS-PAGE was used to separate the proteins, and Coomassie Blue R-250 staining was used to visualize the protein bands (25).

### Label-free LC-MS/MS analysis

LC-MS/MS analysis was performed on a Nanoelute HPLC system (Bruker Daltonics) coupled with a timsTOF Pro mass spectrometer (Bruker) for 60, 120, and 240 min. The mass spectrometer was operated as described in previous studies (26).

### Protein identification and quantification

MaxQuant software (version 1.6.14) and the Swissport_Mus_Musculus_17063_20210106 in Fasta were used to analyze the MS data (27). Trypsin/P was specified as the cleavage enzyme. The maximum number of missed cleavages were 2. Carbamidomethyl (C) was defined as fixed modification, while the oxidation (M) of methionine and the acetylation of the N-terminus of the protein was specified as variable modification. The global false discovery rate (FDR) of peptide and protein identification was <0.01. As for the experimental bias, the calculation of protein abundance was normalized by the spectral protein intensity (LFQ intensity). Proteins with a fold change >1.5 or <0.669 and p value (Student’s *t*-test) <0.05 were considered differentially expressed proteins (28–30).

### Protein functional classification and database search

All differentially expressed proteins’ (DEPs) sequence information was aligned to the *Homo Sapiens* reference sequence (NCBIBLAST-2.2.28+-win32.exe). Blast2GO Command Line was used to complete the annotation from GO terms to proteins. The InterProScan was used to search the EBI database, and it also added functional information of motif to the proteins. The number of DEPs and total proteins correlated to GO terms was compared by Fisher’s exact test to enrich the GO terms, and generate hierarchical clustering heat maps. Fold change >1.5 and the corrected p-value <0.05 is considered significant in GO (31–33).

The Kyoto Encyclopedia of Genes and Genomes (KEGG) pathway enrichment annotation of proteins was performed using the database (https://geneontology.org/). The enrichment of DEPs against all identified proteins were identified by Fisher’s exact test, and a corrected p value <0.05 was considered to be enriched significantly. The annotation of proteins were matched into the database. Online tool KEGG mapper was used to classify these pathways into hierarchical categories.

The protein–protein interaction (PPI) network analysis of the DEPs were searched from IntAct molecular interaction database (https://www.ebi.ac.uk/intact/) or STRING software (https://www.string-db.org/) (version 11.5). The results were downloaded in the XGMML format, and Cytoscape software (https://www.cytoscape.org/, version 3.2.1) was used to visualize and further analyze functional PPI networks (34).

### Real-time reverse transcription-polymerase chain reaction (qRT-PCR)

Total RNA was isolated from liver tissue with TRIzol Reagent (Invitrogen, Carlsbad, CA, United States) and PrimeScript reagent (TaKaRa, Kusatsu, Japan) was used to reverse transcribe into cDNA according to the manufacturer’s instructions. To analyze the target genes’ relative mRNA expression, SYBR Green PCR Master Mix Reagent Kit (Yeasen, Shanghai, China) was used to perform real time qPCR using the Roche 480 detection system. The relative mRNA expression levels were normalized by GAPDH, and 2-△△Ct method was performed to calculate the results. The primer sequences used are listed in **Supplementary Table 2**.

### Western blot analysis

RIPA buffer containing PMSF and phosphatase inhibitor was used to lyse mice liver tissues to extract total protein. After centrifugation at 12000 ×*g* for 15 min, the supernatant was used to measure total protein concentration by BCA method. We used 10% and 12.5% SDS-PAGE gels in the experiment, respectively, based on the molecular weights of the proteins of interest, and then transferred onto a PVDF membrane. The membranes containing proteins were incubated with primary antibodies overnight at 4°C, followed by incubation at room temperature for 1 h with the secondary antibody. The Enhanced Chemiluminescene Plus imaging system was used to detect the protein–antibody immune complexes.

### Antibodies

Anti-FOXO1 antibody (GB11286), Anti-Phospho-FOXO1 antibody (GB113974), Anti-PPAR gamma antibody (GB112205), Anti-SOCS3 antibody (GB113792) and β-actin antibody (GB15003) were purchased from Servicebio Technology (Wuhan, China); Anti-GSTP1(PTM-5992) antibody and Anti-PTPN1(PTM-6344) antibody were obtained from PTM BIO (Suzhou, China); Anti- G0S2 antibody (A9970), β-actin antibody (AC004), β-tubulin antibody and Hsp90α antibody were purchased from ABclonal (Wuhan, China).

### Primary mouse hepatocyte isolation and culture

Primary hepatocytes were isolated from *G0S2* normal expression mice (HFD) and *G0S2* overexpression mice (HFD+G0S2 overexpress) as previously described (35). The isolated primary hepatocytes were cultured in DMEM with 10% fetal bovine serum overnight. After attachment, cells were incubated in Dulbecco’s modified Eagle medium with 0.1 μM insulin or without insulin for 1 h (36).

### Statistical analyses

All data were expressed as the mean ± SD values. Significant differences between the two groups were assessed using an unpaired Student’s t-test, while comparisons among multiple groups were conducted using one-way ANOVA analysis, both performed with GraphPad Prism 8.0. P<0.05 was considered to indicate statistically significant differences. The experiment was repeated three times, using three independent batches of mice and three independent mice in each group.

## Results

### G0S2 increased HFD-induced obesity and insulin resistance

**Figure 1:**
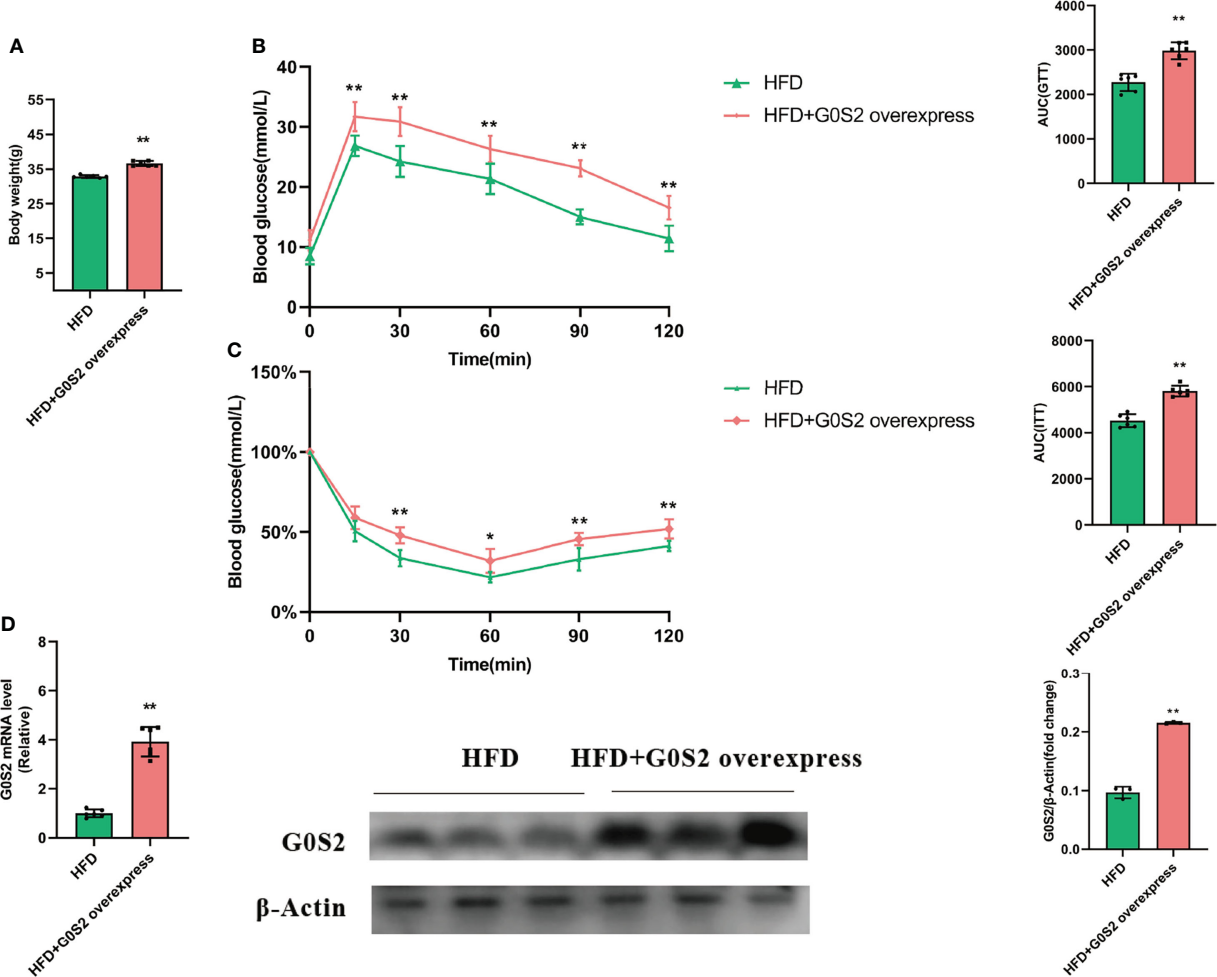
G0S2 increased HFD-induced obesity and insulin resistance. **(A)** Fasting body weight of mice in G0S2 normal expression group (HFD) and G0S2 overexpression group (HFD+G0S2 overexpress). **(B, C)** Representative GTT **(B)** and ITT **(C)** results of mice in the two given groups. **(D)** Western blot and RT-PCR were used to analyze the levels of *G0S2* gene in mice that did and did not receive tail vein injections of Ad-*G0S2* after HFD feeding for 16 weeks in all. *P<0.05; **P<0.01 compared with HFD-vehicle mice.

To address the effects of G0S2 on HFD-fed mice, we injected Ad-*G0S2 in vivo*, directly through the tail vein and continued the HFD for 4 weeks. However, control mice received a vehicle. Compared with control mice, the fasting body weight of *G0S2* overexpression mice was significantly increased (**Figure 1A**). Furthermore, the assays of the GTT and ITT indicated that G0S2 aggravated insulin resistance (**Figures 1B, C**). We examined the hepatic mRNA and protein levels of the *G0S2* gene in both groups by RT-PCR and western blot method. The results showed *G0S2* overexpression of mice upregulation of *G0S2* genes (**Figure 1D**). These data indicate that the *G0S2* overexpression of the mouse model was established successfully.

### *G0S2* overexpression induces differential protein expression in the HFD-diet mouse liver

**Figure 2:**
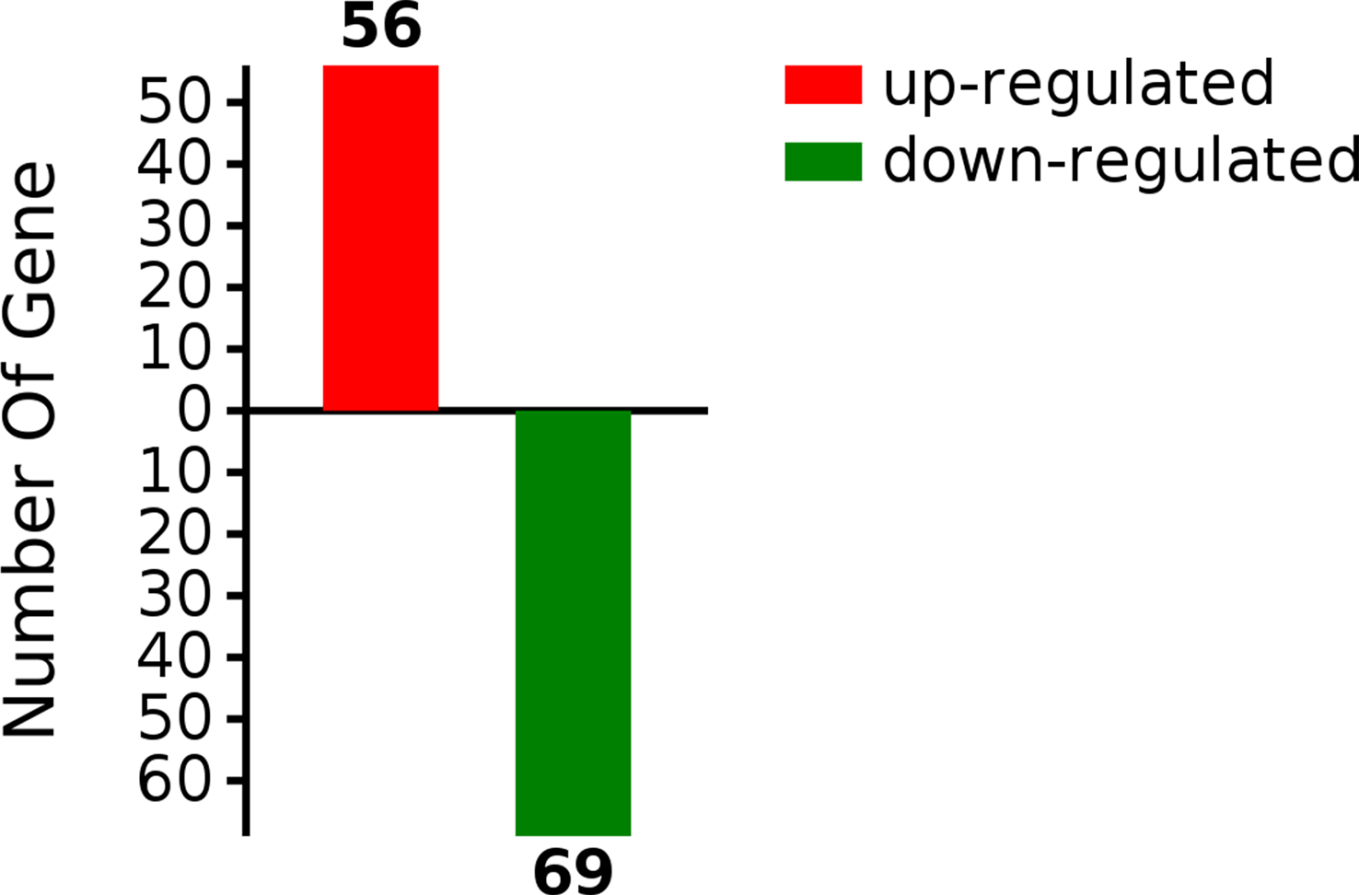
Differentially expressed proteins in the liver tissue of HFD-*G0S2* overexpression mice.

Insulin resistance is strongly associated with NAFLD (16). Deletion of the *G0S2* gene alleviates HFD-induced NAFLD and insulin resistance (13, 37). However, the mechanisms of G0S2 in insulin resistance-related NAFLD are still unknown. To identify the DEPs in the liver of *G0S2* overexpression mice compared to control mice, we performed label-free quantitative proteomics analysis. In all, 3583 proteins were identified by proteomics analysis; among these, 125 proteins were significantly differentially expressed, which included 56 upregulated and 69 downregulated (fold change≥1.5, P<0.05) proteins (**Figure 2**, **Supplementary Table 1**). These results show that G0S2 has an obvious impact on liver protein expression in HFD-diet mice.

### GO analysis

**Figure 3:**
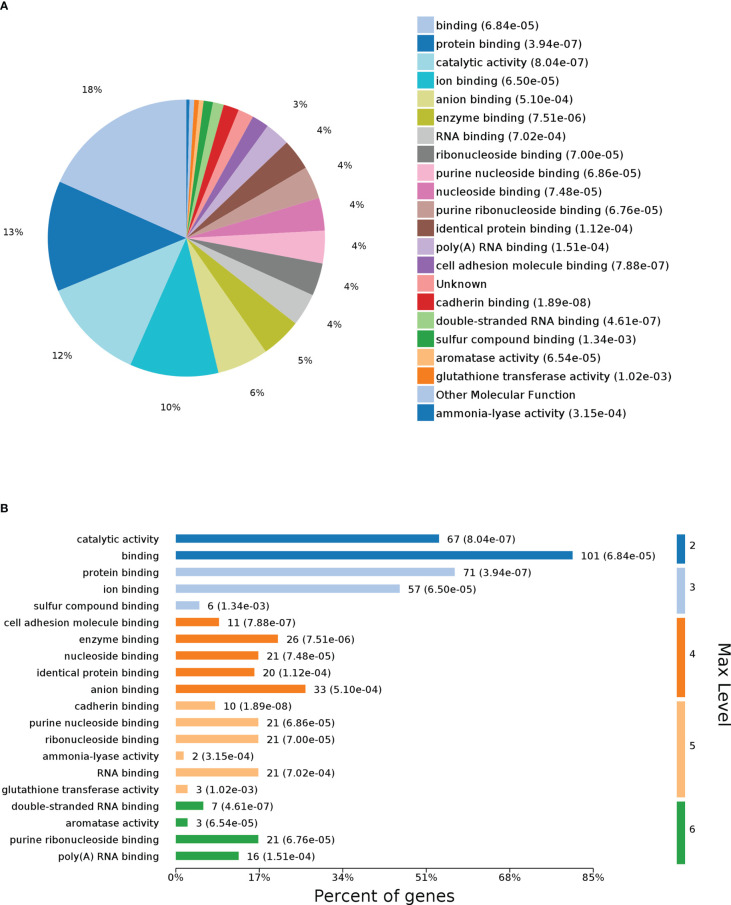
Gene Ontology (GO) enrichment analysis of molecular function for DEPs. **(A)** Pie chart of DEP-enriched GO terms for molecular function (MF). **(B)** Max level for MF.

To further identify the functions of DEPs influenced by G0S2, GO analysis was performed to analyze the proteomics data. The molecular function (MF) category was mainly enriched in “protein binding,” “catalytic activity,” “enzyme binding,” “cell adhesion molecule binding,” and “cadherin binding” (**Figures 3A, B**). These terms suggest a differential influence of G0S2 on NAFLD by interacting with PNPLA2, ABHD5, E-cadherin, and cell adhesions (2, 38–40).

**Figure 4:**
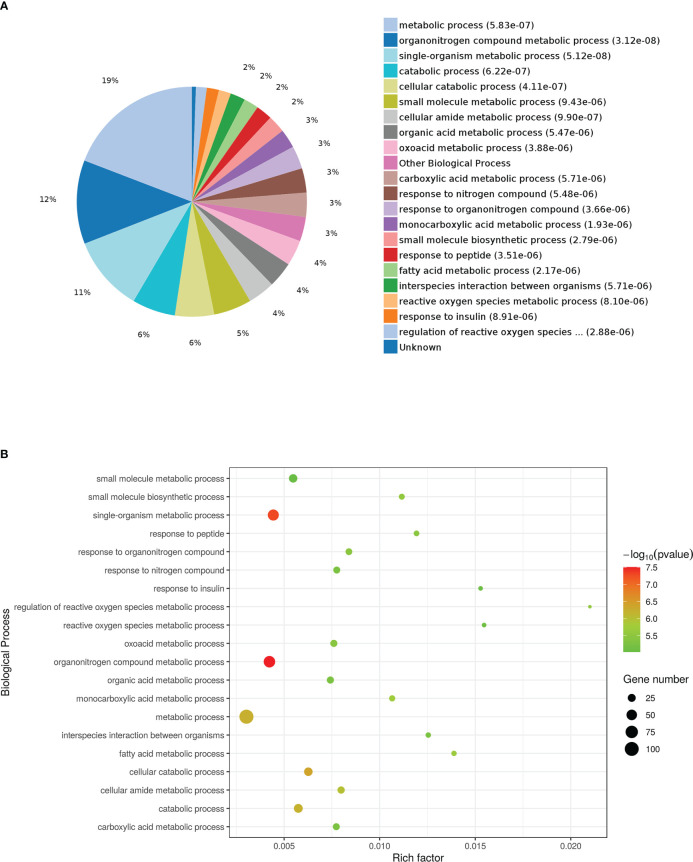
Gene Ontology (GO) enrichment analysis of biological processes for DEPs. **(A)** Pie chart of DEP-enriched GO terms for biological processes (bp). **(B)** Enriched GO terms for bp.

**Figure 5:**
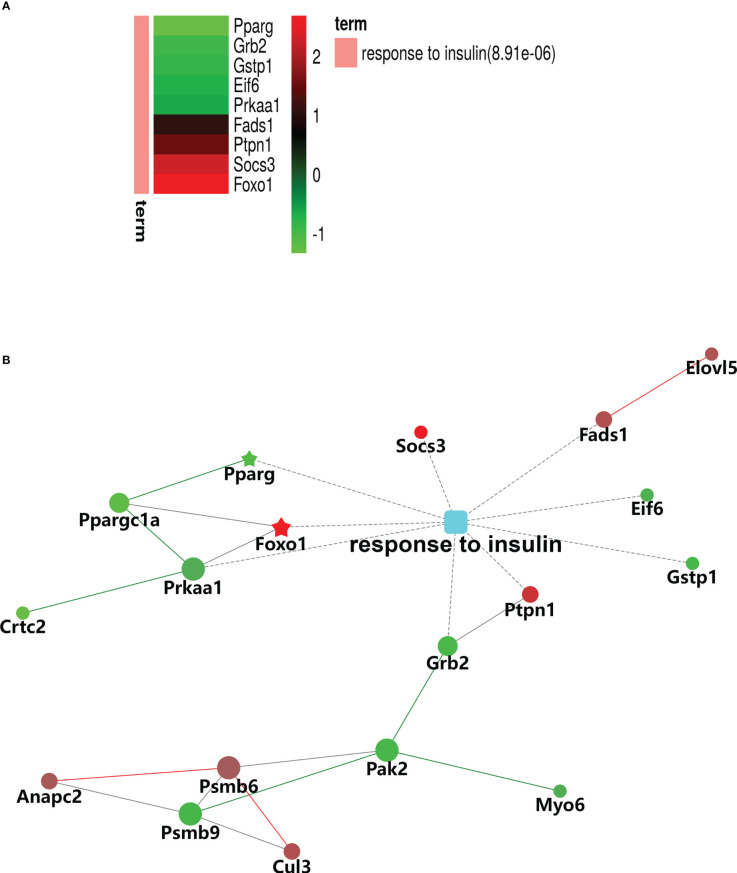
Gene Ontology (GO) enrichment analysis of biological processes for DEPs involved in insulin signaling pathways. **(A)** Heatmap of nine DEPs in response to insulin. **(B)** Protein–protein interaction (PPI) network of DEPs associated with the response to insulin. The red signal represents upregulation and green signal represents downregulation. The red pentagram represents the most pronounced upregulation and green pentagram represents the most pronounced downregulation,.

**Figure 6:**
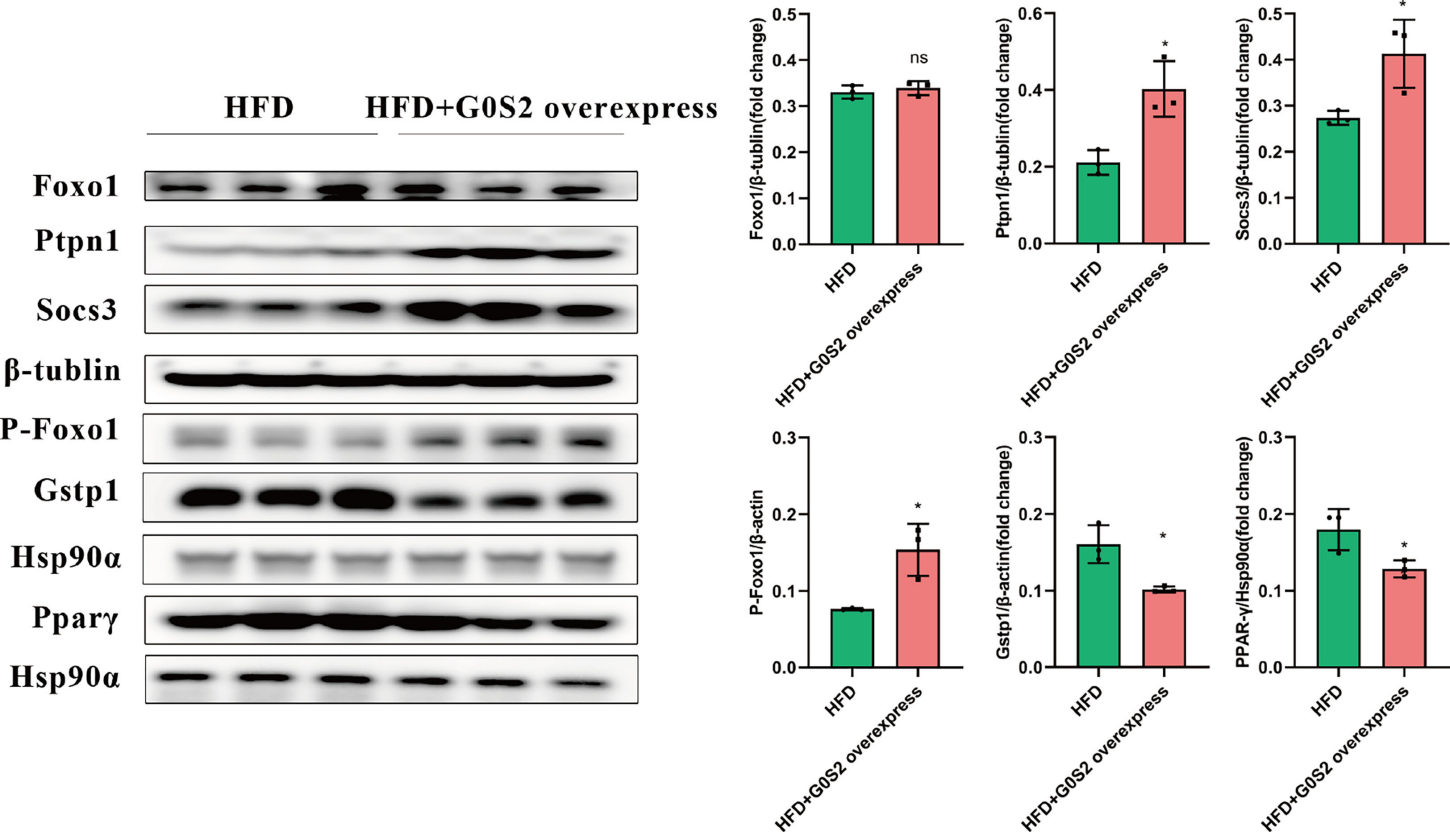
Insulin resistance was evaluated using western blotting and RT-PCR analysis. Upregulation of phosphatase Foxo1, Socs3, and Ptpn1, and downregulation of Gstp1 and PPAR-γ in the livers of HFD-*G0S2* overexpression mice were determined by western blotting and RT-PCR in liver tissue. The experiment was repeated three times, using three independent batches of mice and three independent mice in each group. *P<0.05.

**Figure 7:**
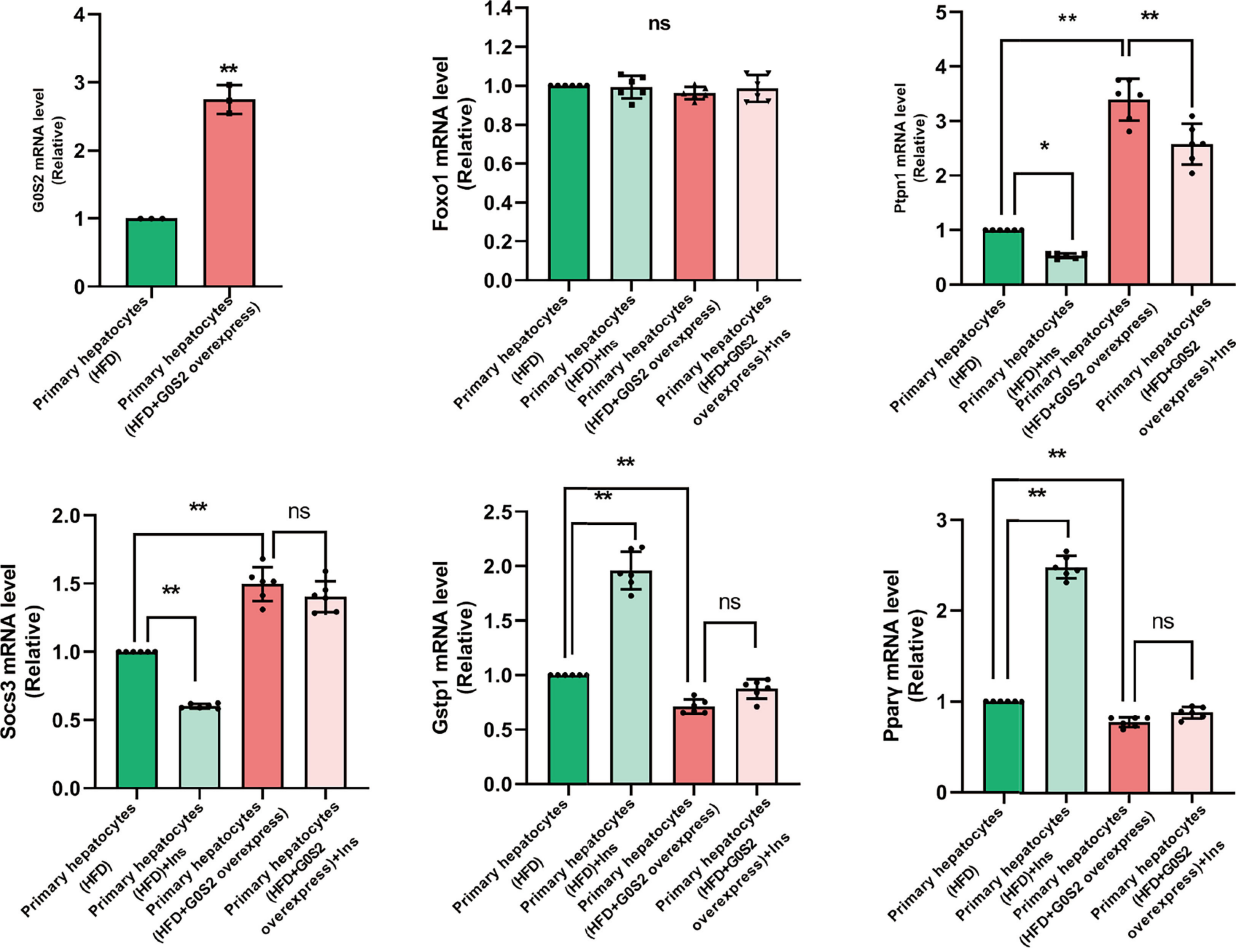
The genes involved in insulin resistance were evaluated using RT-PCR analysis in primary mouse hepatocytes. Downregulation of the phosphatases Foxo1, Socs3, and Ptpn1, as well as upregulation of Gstp1 and PPAR-γ, were determined in primary mouse hepatocytes isolated from *G0S2*-overexpressing mice (HFD+G0S2 overexpression) by RT-PCR. *P<0.05, **P<0.01. ns, P>0.05.

**Figure 8:**
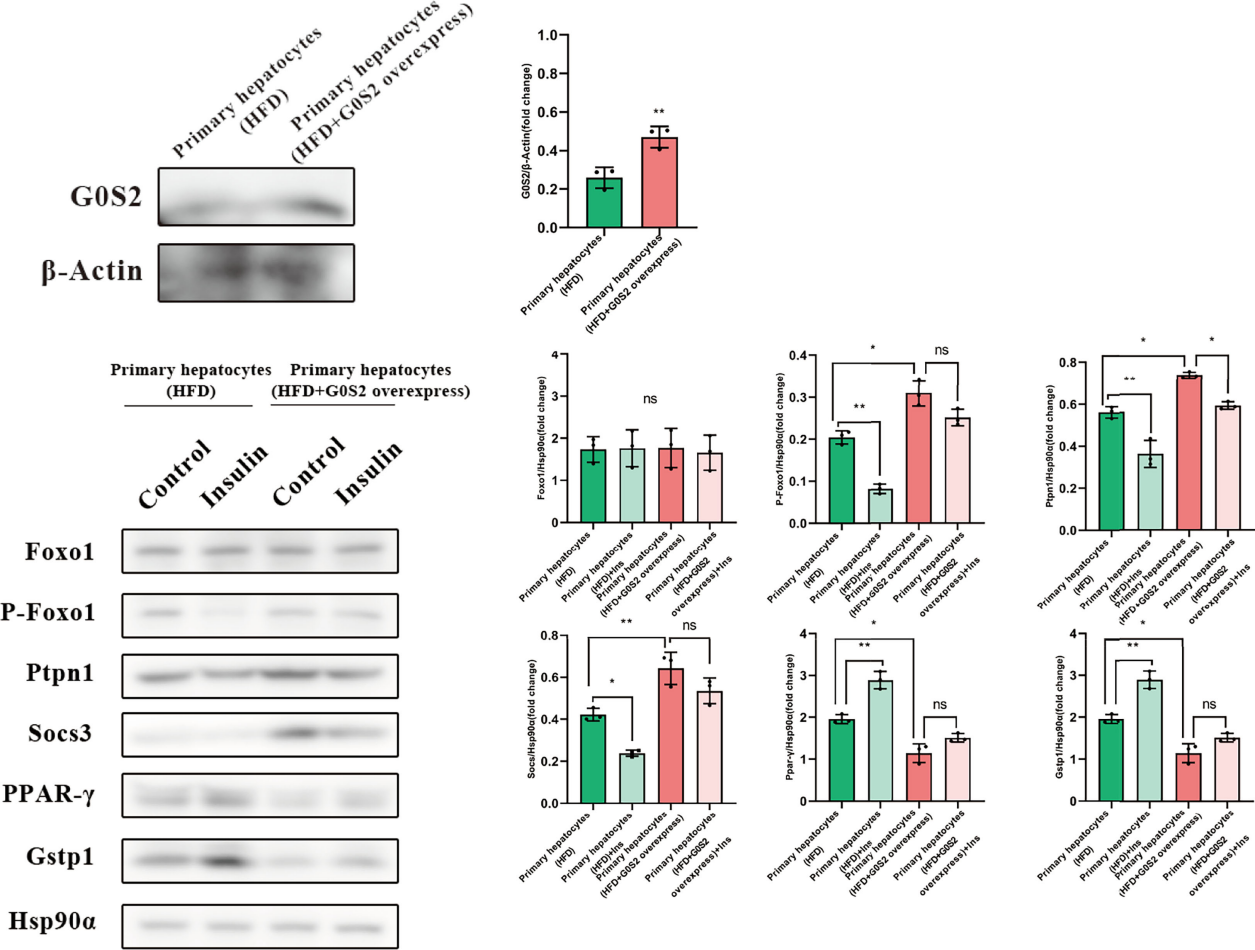
The proteins involved in insulin resistance were evaluated using western blotting analysis in primary mouse hepatocytes. Downregulation of the phosphatases Foxo1, Socs3, and Ptpn1, as well as upregulation of Gstp1 and PPAR-γ, were determined in primary mouse hepatocytes isolated from *G0S2*-overexpressing mice (HFD+G0S2 overexpression) by western blotting. *P<0.05, **P<0.01, ns P>0.05.

The results of the biological process (BP) category showed that 19% of the identified DEPs were enriched in the metabolic process, while 2% of proteins were involved in fatty acid metabolic process and “response to insulin,” respectively (**Figures 4A, B**). Nine DEPs in the liver of *G0S2* overexpression mice were possibly involved in the regulation of insulin homeostasis (four upregulated and five downregulated) (**Figure 5A**, **Table 1**). The results of PPI network analysis showed that G0S2 may interact with Forkhead box protein O1 (Foxo1), Suppressor of cytokine signaling 3 (Socs3), Tyrosine-Protein phosphatase non-receptor type 1 (Ptpn1), Acyl-CoA (8-3)-desaturase (Fads), 5-AMP-activated protein kinase catalytic subunit alpha-1 (Prkaa1), Eukaryotic translation initiation factor 6 (Eif6), Glutathione S-transferase P 1 (Gstp1), Growth factor receptor-bound protein 2 (Grb2), and Peroxisome proliferator-activated receptor gamma (PPAR-γ) (**Figure 5B**). To confirm the effect of DEPs on the regulation of insulin in *G0S2* overexpression mice, five DEPs were validated using WB assay. Consistent with the results of the proteomics analysis, an obvious increase of phosphatase Foxo1, Socs3, and Ptpn1, and an obvious decrease of Gstp1 and PPAR-γ was observed (**Figure 6**). Next, primary mouse hepatocytes isolated from mice with normal G0S2 expression (HFD) and mice overexpressing G0S2 (HFD+G0S2 overexpression) were either stimulated with insulin or left unstimulated. These DEPs were differentially regulated under basal and insulin-stimulated (0.1μM, 1h) conditions. Downregulation of phosphatase Foxo1, Socs3, and Ptpn1, and upregulation of Gstp1 and PPAR-? in the livers of HFD-G0S2 overexpression mice were determined by western blotting and RT-PCR in primary mouse hepatocytes (**Figures 7**, **8**) and suggest that G0S2 plays an important role in the regulation of insulin sensitivity.

**Table 1:** Identification of *G0S2* overexpression-induced differentially expressed proteins associated with the response to insulin.

Change	Protein IDs	Protein Name	Gene Name	Fold Change
up	Q9R1E0	Forkhead box protein O1	Foxo1	6.420271268
up	O35718	Suppressor of cytokine signaling 3	Socs3	4.792348761
up	P35821	Tyrosine-protein phosphatase non-receptor type 1	Ptpn1	2.911627141
up	Q920L1	Acyl-CoA (8-3)-desaturase	Fads1	2.059835232
down	Q5EG47	5-AMP-activated protein kinase catalytic subunit alpha-1	Prkaa1	0.650331086
down	O55135	Eukaryotic translation initiation factor 6	Eif6	0.616771107
down	P19157	Glutathione S-transferase P 1	Gstp1	0.563400004
down	Q60631	Growth factor receptor-bound protein 2	Grb2	0.526383608
down	P37238	Peroxisome proliferator-activated receptor gamma	Pparg	0.397358829

Up, upregulated; down, downregulated.

### KEGG analysis of DEPs

**Figure 9:**
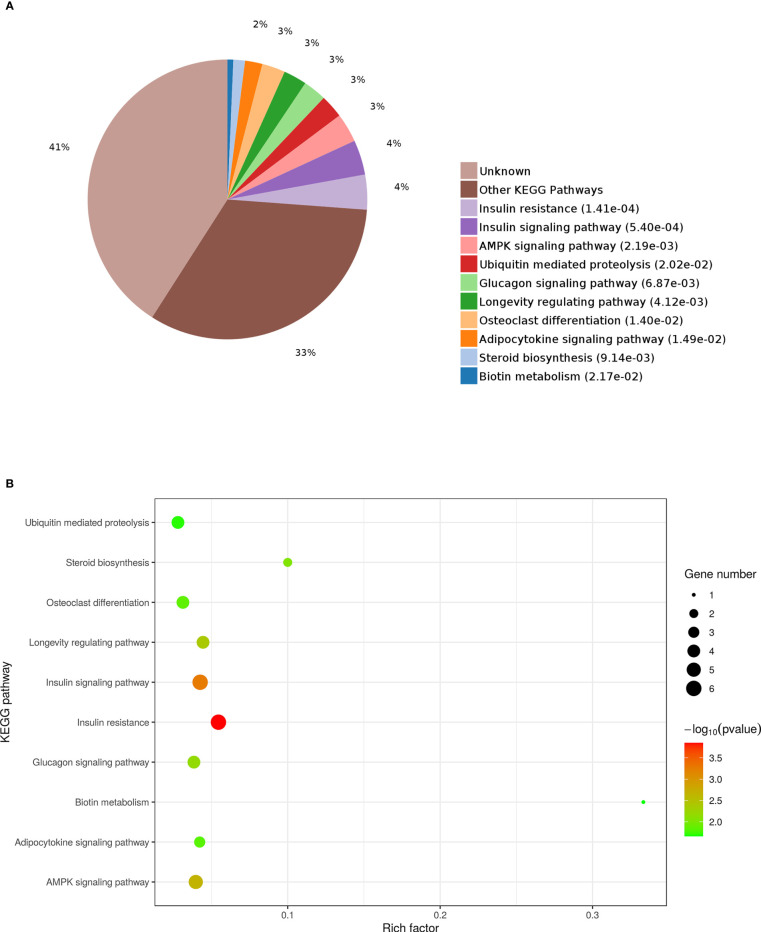
Kyoto Encyclopedia of Genes and Genomes (KEGG) pathway analysis of DEPs. **(A)** Pie chart of DEP-enriched KEGG pathways. **(B)** KEGG pathway enrichment distribution of DEPs.

**Figure 10:**
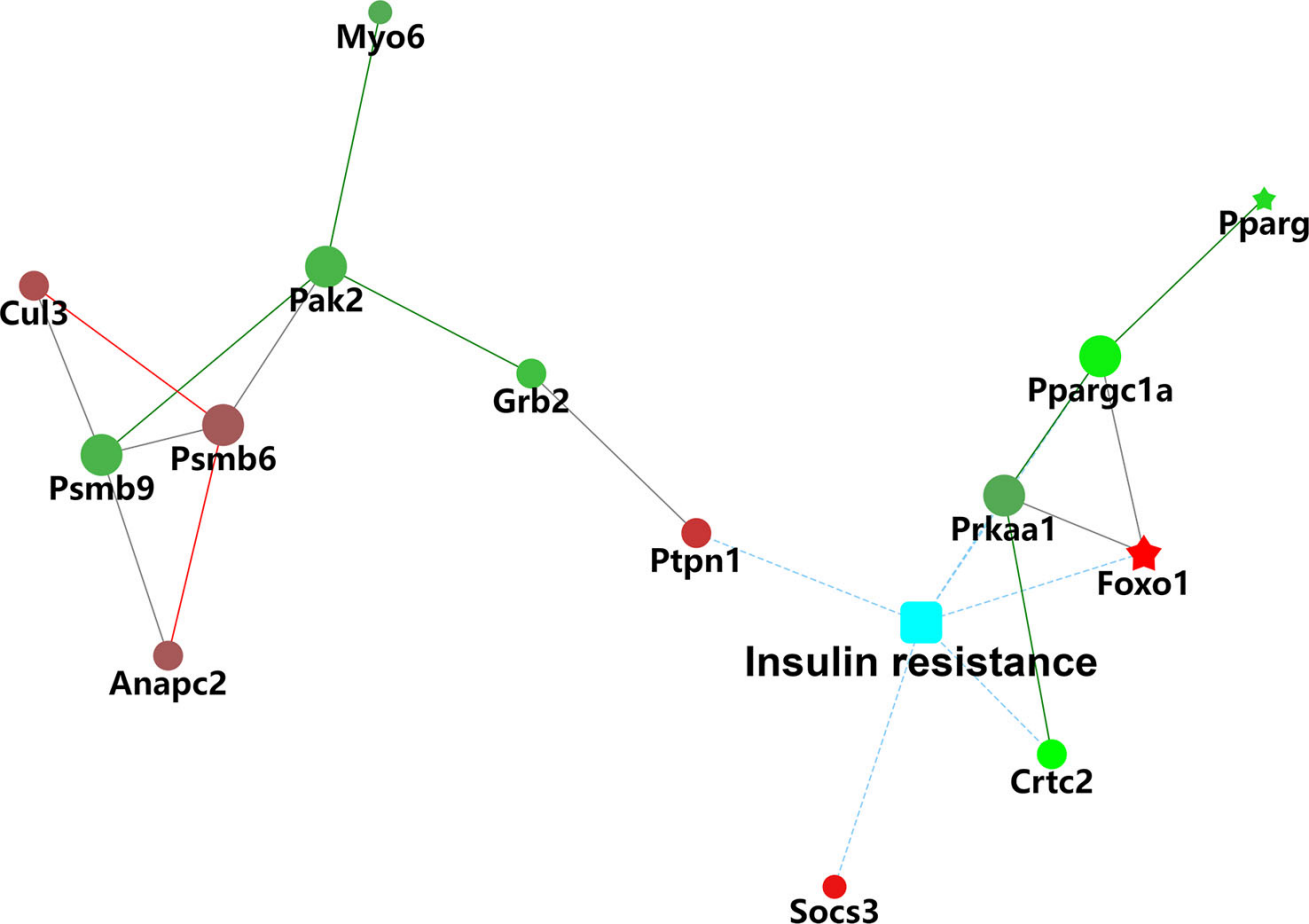
Protein–protein interaction (PPI) network analysis of differentially insulin resistance-associated proteins.

KEGG enrichment analysis was used to further explore the functions of the identified DEPs. The results revealed that the enrichment of DEPs in the pathways were associated with insulin resistance (4%), insulin signaling pathway (4%), and AMPK signaling pathway (3%). Additionally, 3%, 3%, and 2% of DEPs were associated with “Glucagon signaling pathway,” “Adipocytokine signaling pathway,” and “Steroid biosynthesis” (**Figures 9A, B**). To better understand the relationship between the nine DEPs and insulin resistance, another network of PPI was established (**Figure 10**). The complicated network comprised various insulin resistance-associated proteins, which was interacted with each other, suggesting that G0S2 might be the key factor in regulating insulin sensitivity.

## Discussion

G0S2 is primarily a cell cycle-regulated protein that was originally identified in blood mononuclear cells and has 78% homology between mouse and human isoforms (2). A further study ruled out that G0S2 is involved in various biological and pathological processes such as glycolipid metabolism, inflammation, immunization, and cancer (41–44).

Increasing research indicates that interfering hepatic G0S2 expression represents an effective change in the level of hepatic TG and blood glucose (21, 35). G0S2 knockout mice exhibit a lower level of hepatic triglycerides and were resistant to HFD-induced liver steatosis (12). Moreover, clinical trials show that the mRNA and protein content of G0S2 are reduced in poorly controlled type 1 and type 2 diabetic subjects (41, 45). These previous studies suggested that G0S2 is critical for the regulation of physiological and pathological processes of NAFLD and diabetes.

Accumulating studies support that insulin resistance is one of the earliest manifestations of a constellation of metabolic disease, including T2DM and NAFLD (46). Some extracellular factors lead to defects in the responsiveness of cells to insulin, such as lipids and other circulating factors that perturb the intracellular concentration of ceramide (14). Insulin resistance is the main risk factor of diabetes and NAFLD (11, 47, 48). However, the mechanisms of G0S2 regulated NAFLD and diabetes is still not clearly known.

In our study, the protein expression in the livers of G0S2-overexpression mice was analyzed by label-free LC-MS/MS quantitative proteomics. The results of proteomics demonstrated that there were four upregulated proteins that were related to insulin signaling pathways. Foxo1 was mainly involved in insulin resistance and lipid metabolism. Previous studies have revealed that Foxo1 participates in insulin resistance and β-cell failure in T2DM patients and leads to gluconeogenesis dysfunction and cell apoptosis. However, inhibition of Foxo1 improves insulin resistance (49, 50). However, some studies show that inhibition of Foxo1 interacts with ATGL leading to hepatic steatosis (51). Our study showed that Foxo1 was upregulated by 6.4-fold and was a pro-insulin resistance protein. Hence, the above research results suggest that G0S2 exerts an important role in regulating the insulin signaling pathway in the liver.

The suppressor of cytokine signaling (SOCS) family of proteins are negative regulators of cytokine signaling. The expression of Socs3 in the liver, skeletal muscle, and adipose tissue is upregulated in obese rodents (52, 53). In obese patients with NAFLD, the abundance of Socs3 in mononuclear cells was also increased (54, 55). In an Socs3 AKO mouse model, the HFD increased the levels of Socs3 in adipose tissue of WT mice; however, Socs3 AKO mice failed to show the same results (56). Socs3 has been shown to play an important role in insulin sensitivity, because it inhibits tyrosine phosphorylation of the relevant receptor, such as insulin receptor and insulin receptor substrate-1 (IRS1) (57, 58). A recent study found that Polygoni Cuspidati ethanol extract attenuates obesity, NAFLD, and IR *via* inhibitions of Socs3 (59). The findings of our study suggest that upregulation of G0S2 induced impairment of insulin signaling. Insulin resistance is likely an important determinant of the negative effects of G0S2 targeting NAFLD and diabetes.

*Ptpn1*, the gene coding for Protein Tyrosine Phosphatase-1B, plays a critical role in negative regulation of insulin signaling. The upregulation of Ptpn1 in tissues and cells inactivates protein tyrosine kinase (PTK), blocks the effect of insulin on binding to insulin receptors and dephosphorylation of tyrosine residues on insulin receptors substrates, leading to insulin resistance and finally to diabetes (60–62). A study revealed that by inhibiting Ptpn1 expression and promoting phosphorylation of insulin receptor, microRNA-206 impaired hepatic lipogenesis and exerted the beneficial effect of preventing hepatic steatosis (63). Our study results are consistent with the above observations in that it suggests that inhibition of Ptpn1 expression mediates the beneficial effect of G0S2 on NAFLD and diabetes.

Our study results demonstrated that the levels of Gstp1 and PPAR-γ were significantly down-regulated after overexpression of G0S2. Previous studies ruled out that Gstp1 is closely involved in the inhibition of cell apoptosis and regulation of cell oxidative stress (64, 65). The tumor necrosis factor-related receptor 2 (TRAF2) interacts with apoptosis signal regulating kinase 1 (ASK1), and the interaction between them could be abolished by binding Gstp1 to TRAF2 (66). Gstp1 regulated the ASK1-MEK-JNK/p38 pathway negatively and inhibited cell apoptosis (67). Another research on humans showed that participants with Gstp1 AG genotypes showed stronger associations between insulin resistance markers who were exposed to air pollution (68).

PPAR agonists, lipid sensors that modulate whole-body energy metabolism, have been used to treat dyslipidemia and diabetes for decades. PPAR-γ increases systemic insulin sensitivity by increasing adipocyte differentiation and fatty acid uptake and storage in lipid droplets (69). PPAR-γ deficiency in adipose tissue causes metabolic dysfunction in mice (70). Under conditions of energy deficiency, PPAR-γ on Lys 268 and Lys 293 was deacetylated by SIRT 1. Regulation of PPAR-γ can protect mice from HFD-induced insulin resistance (71–73). Notably, thermogenesis was enhanced in the mouse model of Kdm2a deficiency in macrophages, and the obesity induced by HFD was prevented by enhancing H3K36me2 at the PPAR-γ locus. The upregulation of PPAR-γ may highlight a new mechanism by which G0S2 helps improve insulin sensitivity in NAFLD and diabetes.

There are some limitations to this study. For example, there was no control group of mice on normal chow diet. Based on the absence of these groups as control, the results of our study should be interpreted with caution, and further investigations are needed. Another limitation is that we did not test the effects of G0S2 gene deletion to determine whether such deletion is sufficient to improve insulin resistance.

In conclusion, we focused our study on the effect of G0S2 on insulin resistance. Insulin resistance is a key contributor to the pathogenesis of NAFLD, diabetes, and fatty and other metabolic diseases. Our research demonstrates that the expression patterns of several proteins associated with insulin signaling pathway are consistent with the change of insulin resistance after overexpression of G0S2. These observations might uncover the molecular mechanisms of metabolic diseases and provide novel insights into potential therapeutic targets for NAFLD, diabetes, and other metabolic diseases.

## Data availability statement

The datasets presented in this study can be found in online repositories. The names of the repository/repositories and accession number(s) can be found in the article/**Supplementary Material**.

## Ethics statement

All methods are implemented in conformity with relative instructions and regulations. All surgeries were performed under sodium pentobarbital anesthesia to minimize pain. The present study was approved by the Ethics Committee of Shandong Provincial Hospital (NSFC: NO.2019-131). All methods were performed following the ARRIVE guidelines.

## Author contributions

DW was the experimental designer and executor of the experimental study, completed the data analysis, and wrote the first draft of the paper. SM was the conceptualizer and leader of the project, and directed the experimental design, data analysis, and paper writing and revision. ZZ, WS, YY, and MJ contributed to experiment and data analyze. All authors contributed to the article and approved the submitted version.

## References

[1] Wang X Liu M Zhang J Brown NK Zhang P Zhang Y. CD24-siglec axis is an innate immune checkpoint against metaflammation and metabolic disorder. Cell Metab (2022) 34(8):1088–103. doi: 10.1016/j.cmet PMC9393047 35921817

[2] Eckel RH Grundy SM Zimmet PZ. The metabolic syndrome. Lancet (2005) 365(9468):1415–28. doi: 10.1016/S0140-6736(05)66378-7 15836891

[3] Goga A Stoffel M. Therapeutic RNA-silencing oligonucleotides in metabolic diseases. Nat Rev Drug Discovery (2022) 21(6):417–39. doi: 10.1038/s41573-022-00407-5 35210608

[4] Samuel VT Shulman GI. Mechanisms for insulin resistance: Common threads and missing links. Cell (2012) 148(5):852–71. doi: 10.1016/j.cell PMC3294420 22385956

[5] Kioka H Kato H Fujikawa M Tsukamoto O Suzuki T Imamura H. Evaluation of intramitochondrial ATP levels identifies G0/G1 switch gene 2 as a positive regulator of oxidative phosphorylation. Proc Natl Acad Sci (2013) 111(1):273–8. doi: 10.1073/pnas.1318547111 PMC3890790 24344269

[6] Zhang X Heckmann BL Campbell LE Liu J. G0S2: A small giant controller of lipolysis and adipose-liver fatty acid flux. Biochim Biophys Acta (BBA) - Mol Cell Biol Lipids (2017) 1862(10):1146–54. doi: 10.1016/j.bbalip.2017.06.007 PMC5890940 28645852

[7] Ivanovic Z Yamada T Park CS Burns A Nakada D Lacorazza HD. The cytosolic protein G0S2 maintains quiescence in hematopoietic stem cells. PloS One (2012) 7(5):e38280. doi: 10.1371/journal.pone.0038280 22693613 PMC3365016

[8] Kitada M Koya D. Autophagy in metabolic disease and ageing. Nat Rev Endocrinol (2021) 17(11):647–61. doi: 10.1038/s41574-021-00551-9 34508250

[9] Targher G Lonardo A Byrne CD. Nonalcoholic fatty liver disease and chronic vascular complications of diabetes mellitus. Nat Rev Endocrinol (2017) 14(2):99–114. doi: 10.1038/nrendo.2017.173 29286050

[10] Mantovani A Scorletti E Mosca A Alisi A Byrne CD Targher G. Complications, morbidity and mortality of nonalcoholic fatty liver disease. Metabolism (2020) 111S:154170. doi: 10.1016/j.metabol.2020.154170 32006558

[11] Targher G Corey KE Byrne CD Roden M. The complex link between NAFLD and type 2 diabetes mellitus — mechanisms and treatments. Nat Rev Gastroenterol Hepatol (2021) 18(9):599–612. doi: 10.1038/s41575-021-00448-y 33972770

[12] Ma Y Zhang M Yu H Lu J Cheng KKY Zhou J. Activation of G0/G1 switch gene 2 by endoplasmic reticulum stress enhances hepatic steatosis. Metabolism (2019) 99:32–44. doi: 10.1016/j.metabol.2019.06.015 31271806

[13] El-Assaad W El-Kouhen K Mohammad AH Yang J Morita M Gamache I. Deletion of the gene encoding G0/G1 switch protein 2 (G0s2) alleviates high-fat-diet-induced weight gain and insulin resistance, and promotes browning of white adipose tissue in mice. Diabetologia (2015) 58(1):149–57. doi: 10.1007/s00125-014-3429-z PMC5001162 25381555

[14] Irimia JM Meyer CM Segvich DM Surendran S DePaoli-Roach AA Morral N. Lack of liver glycogen causes hepatic insulin resistance and steatosis in mice. J Biol Chem (2017) 292(25):10455–64. doi: 10.1074/jbc.M117.786525 PMC5481557 28483921

[15] Loomba R Abraham M Unalp A Wilson L Lavine J Doo E. Association between diabetes, family history of diabetes and risk of nonalcoholic steatohepatitis and fibrosis. Hepatology (2012) 56(3):943–51. doi: 10.1002/hep.25772 PMC3407289 22505194

[16] Valenti L Bugianesi E Pajvani U Targher G. Nonalcoholic fatty liver disease: cause or consequence of type 2 diabetes? Liver Int (2016) 36(11):1563–79. doi: 10.1111/liv.13185 27276701

[17] Donnelly KL Smith CI Schwarzenberg SJ Jessurun J Boldt MD Parks EJ. Sources of fatty acids stored in liver and secreted *via* lipoproteins in patients with nonalcoholic fatty liver disease. J Clin Invest (2005) 115(5):1343–51. doi: 10.1172/jci200523621 PMC1087172 15864352

[18] Ferguson D Finck BN. Emerging therapeutic approaches for the treatment of NAFLD and type 2 diabetes mellitus. Nat Rev Endocrinol (2021) 17(8):484–95. doi: 10.1038/s41574-021-00507-z PMC8570106 34131333

[19] Zhang W Bu So Y Mashek Mara T O-Sullivan I Sibai Z Khan Salmaan A. The aetiology and molecular landscape of insulin resistance. Cell Rep (2016) 15(2):349–59. doi: 10.1016/j.celrep.2016.03.021 PMC5346032 27050511

[20] Heckmann BL Zhang X Saarinen AM Schoiswohl G Kershaw EE Zechner R. Liver X receptor α mediates hepatic triglyceride accumulation through upregulation of G0/G1 switch gene 2 expression. JCI Insight (2017) 2(4):e88735. doi: 10.1172/jci.insight.88735 28239648 PMC5313069

[21] Jaeger D Schoiswohl G Hofer P Schreiber R Schweiger M Eichmann TO. Fasting-induced G0/G1 switch gene 2 and FGF21 expression in the liver are under regulation of adipose tissue derived fatty acids. J Hepatol (2015) 63(2):437–45. doi: 10.1016/j.jhep.2015.02.035 PMC4518503 25733154

[22] Nojima K Sugimoto K Ueda H Babaya N Ikegami H Rakugi H. Analysis of hepatic gene expression profile in a spontaneous mouse model of type 2 diabetes under a high sucrose diet. Endocr J (2012) 60:261–74. doi: 10.1507/endocrj 23131898

[23] Wu D Wang X Han Y Wang Y. The effect of lipocalin-2 (LCN2) on apoptosis: a proteomics analysis study in an LCN2 deficient mouse model. BMC Genomics (2021) 22:892. doi: 10.1186/s12864-021-08211-y 34903175 PMC8670060

[24] Wiśniewski JR Zougman A Nagaraj N Mann M. Universal sample preparation method for proteome analysis. Nat Methods (2009) 6(5):359–62. doi: 10.1038/nmeth.1322 19377485

[25] Rashid ST Humphries JD Byron A Dhar A Askari JA Selley JN. Proteomic analysis of extracellular matrix from the hepatic stellate cell line LX-2 identifies CYR61 and wnt-5a as novel constituents of fibrotic liver. J Proteome Res (2012) 11(8):4052–64. doi: 10.1021/pr3000927 PMC3411196 22694338

[26] Levin Y Schwarz E Wang L Leweke FM Bahn S. Label-free LC-MS/MS quantitative proteomics for large-scale biomarker discovery in complex samples. J Separation Sci (2007) 30(14):2198–203. doi: 10.1002/jssc.200700189 17668910

[27] Cox J Mann M. MaxQuant enables high peptide identification rates, individualized p.p.b.-range mass accuracies and proteome-wide protein quantification. Nat Biotechnol (2008) 26(12):1367–72. doi: 10.1038/nbt.1511 19029910

[28] Zybailov B Coleman KM Florens L Washburn PM. Correlation of relative abundance ratios derived from peptide ion chromatograms and spectrum counting for quantitative proteomic analysis using stable isotope labeling. Anal Chem (2005) 77:6218–24. doi: 10.1021/ac050846r 16194081

[29] Pinto SM Manda SS Kim M-S Taylor K Selvan LDN Balakrishnan L. Functional annotation of proteome encoded by human chromosome 22. J Proteome Res (2014) 13(6):2749–60. doi: 10.1021/pr401169d PMC4059257 24669763

[30] Toraskar J Magnussen SN Hagen L Sharma A Hoang L Bjørkøy G. A novel truncated form of nephronectin is present in small extracellular vesicles isolated from 66cl4 cells. J Proteome Res (2019) 18(3):1237–47. doi: 10.1021/acs.jproteome.8b00859 30707844

[31] Gotz S Garcia-Gomez JM Terol J Williams TD Nagaraj SH Nueda MJ. High-throughput functional annotation and data mining with the Blast2GO suite. Nucleic Acids Res (2008) 36(10):3420–35. doi: 10.1093/nar/gkn176 PMC2425479 18445632

[32] Wang X Shi Y He R Li B Huang A. Label-free quantitative proteomic analysis of the biological functions of moringa oleifera seed proteins provides insights regarding the milk-clotting proteases. Int J Biol Macromol (2020) 144:325–33. doi: 10.1016/j.ijbiomac.2019.12.070 31830451

[33] Jin Y Li D Sun T Du Y Gao Y Ding R. Pathological features of enterovirus 71-associated brain and lung damage in mice based on quantitative proteomic analysis. Front Microbiol (2021) 12:663019. doi: 10.3389/fmicb.2021.663019 34220748 PMC8249819

[34] Shannon P Markiel A Ozier O Baliga NS Wang JT Ramage D. Cytoscape: A software environment for integrated models of biomolecular interaction networks. Genome Res (2003) 13(11):2498–504. doi: 10.1101/gr.1239303 PMC403769 14597658

[35] Zhao Z-H Wang Z-X Zhou D Han Y Ma F Hu Z. Sodium butyrate supplementation inhibits hepatic steatosis by stimulating liver kinase B1 and insulin-induced gene. Cell Mol Gastroenterol Hepatol (2021) 12(3):857–71. doi: 10.1016/j.jcmgh.2021.05.006 PMC8346675 33989817

[36] Yan H Yang W Zhou F Li X Pan Q Shen Z. Estrogen improves insulin sensitivity and suppresses gluconeogenesis *via* the transcription factor Foxo1. Diabetes (2019) 68(2):291–304. doi: 10.2337/db18-0638 30487265 PMC6341301

[37] Zhang X Xie X Heckmann BL Saarinen AM Czyzyk TA Liu J. Targeted disruption of G0/G1 switch gene 2 enhances adipose lipolysis, alters hepatic energy balance, and alleviates high-fat diet– induced liver steatosis. Diabetes (2014) 63(3):934–46. doi: 10.2337/db13-1422 PMC3931401 24194501

[38] Hatsell S Rowlands T Hiremath M Cowin P. β-catenin and tcfs in mammary development and cancer. J Mammary Gland Biol Neoplasia (2003) 8:145–58. doi: 10.1023/A:1025944723047 14635791

[39] Yamaguchi T Osumi T. Chanarin–dorfman syndrome: Deficiency in CGI-58, a lipid droplet-bound coactivator of lipase. Biochim Biophys Acta (BBA) - Mol Cell Biol Lipids (2009) 1791(6):519–23. doi: 10.1016/j.bbalip.2008.10.012 19061969

[40] Lass A Zimmermann R Haemmerle G Riederer M Schoiswohl G Schweiger M. Adipose triglyceride lipase-mediated lipolysis of cellular fat stores is activated by CGI-58 and defective in chanarin-dorfman syndrome. Cell Metab (2006) 3(5):309–19. doi: 10.1016/j.cmet.2006.03.005 16679289

[41] Voss TS Vendelbo MH Kampmann U Pedersen SB Nielsen TS Johannsen M. Substrate metabolism, hormone and cytokine levels and adipose tissue signalling in individuals with type 1 diabetes after insulin withdrawal and subsequent insulin therapy to model the initiating steps of ketoacidosis. Diabetologia (2018) 62(3):494–503. doi: 10.1007/s00125-018-4785-x 30506451

[42] Ai K Pan J Zhang P Li H He Z Zhang H. Methyl-CpG-binding domain protein 2 contributes to renal fibrosis through promoting polarized M1 macrophages. Cell Death Dis (2022) 13(2):125. doi: 10.1038/s41419-022-04577-3 35136032 PMC8826408

[43] Xu L Li Z Li Y Luo Z Luo Y Xiao B. The expression pattern and regulatory mechanism of the G0/G1 switch gene 2 (G0S2) in the pathogenesis and treatment ofAChR myasthenia gravis (MG). Mediators Inflamm (2020) 2020:1–11. doi: 10.1155/2020/4286047 PMC7545457 33061827

[44] Mohan DR Lerario AM Else T Mukherjee B Almeida MQ Vinco M. Targeted assessment of G0S2 methylation identifies a rapidly recurrent, routinely fatal molecular subtype of adrenocortical carcinoma. Clin Cancer Res (2019) 25(11):3276–88. doi: 10.1158/1078-0432.Ccr-18-2693 PMC7117545 30770352

[45] Nielsen TS Kampmann U Nielsen RR Jessen N Ørskov L Pedersen SB. Reduced mRNA and protein expression of perilipin a and G0/G1 switch gene 2 (G0S2) in human adipose tissue in poorly controlled type 2 diabetes. J Clin Endocrinol Metab (2012) 97(7):E1348–52. doi: 10.1210/jc.2012-1159 22535977

[46] Younossi ZM Golabi P de Avila L Paik JM Srishord M Fukui N. The global epidemiology of NAFLD and NASH in patients with type 2 diabetes: A systematic review and meta-analysis. J Hepatol (2019) 71(4):793–801. doi: 10.1016/j.jhep.2019.06.021 31279902

[47] Dong B Zhou Y Wang W Scott J Kim K Sun Z. Vitamin d receptor activation in liver macrophages ameliorates hepatic inflammation, steatosis, and insulin resistance in mice. Hepatology (2020) 71(5):1559–74. doi: 10.1002/hep.30937 31506976

[48] Rohm TV Meier DT Olefsky JM Donath MY. Inflammation in obesity, diabetes, and related disorders. Immunity (2022) 55(1):31–55. doi: 10.1016/j.immuni.2021.12.013 35021057 PMC8773457

[49] Kitamura T. The role of FOXO1 in β-cell failure and type 2 diabetes mellitus. Nat Rev Endocrinol (2013) 9(10):615–23. doi: 10.1038/nrendo.2013.157 23959366

[50] Xing Y-Q Li A Yang Y Li X-X Zhang L-N Guo H-C. The regulation of FOXO1 and its role in disease progression. Life Sci (2018) 193:124–31. doi: 10.1016/j.lfs.2017.11.030 29158051

[51] Chen G Yu D Nian X Liu J Koenig RJ Xu B. LncRNA SRA promotes hepatic steatosis through repressing the expression of adipose triglyceride lipase (ATGL). Sci Rep (2016) 6(1):35531. doi: 10.1038/srep35531 27759039 PMC5069493

[52] Ueki K Kondo T Kahn CR. Suppressor of cytokine signaling 1 (SOCS-1) and SOCS-3 cause insulin resistance through inhibition of tyrosine phosphorylation of insulin receptor substrate proteins by discrete mechanisms. Mol Cell Biol (2004) 24(12):5434–46. doi: 10.1128/mcb.24.12.5434-5446.2004 PMC419873 15169905

[53] Steinberg GR Smith AC Wormald S Malenfant P Collier C Dyck DJ. Endurance training partially reverses dietary-induced leptin resistance in rodent skeletal muscle. Am J Physiol Endocrinol Metab (2004) 286(1):E57–63. doi: 10.1152/ajpendo.00302.2003 14662513

[54] Ghanim H Aljada A Daoud N Deopurkar R Chaudhuri A Dandona P. Role of inflammatory mediators in the suppression of insulin receptor phosphorylation in circulating mononuclear cells of obese subjects. Diabetologia (2006) 50(2):278–85. doi: 10.1007/s00125-006-0508-9 17180352

[55] Ghanim H Abuaysheh S Sia CL Korzeniewski K Chaudhuri A Fernandez-Real JM. Increase in plasma endotoxin concentrations and the expression of toll-like receptors and suppressor of cytokine signaling-3 in mononuclear cells after a high-fat, high-carbohydrate meal. Diabetes Care (2009) 32(12):2281–7. doi: 10.2337/dc09-0979 PMC2782991 19755625

[56] Palanivel R Fullerton MD Galic S Honeyman J Hewitt KA Jorgensen SB. Reduced Socs3 expression in adipose tissue protects female mice against obesity-induced insulin resistance. Diabetologia (2012) 55(11):3083–93. doi: 10.1007/s00125-012-2665-3 PMC5233443 22872213

[57] Senn JJ Klover PJ Nowak IA Zimmers TA Koniaris LG Furlanetto RW. Suppressor of cytokine signaling-3 (SOCS-3), a potential mediator of interleukin-6-dependent insulin resistance in hepatocytes. J Biol Chem (2003) 278(16):13740–6. doi: 10.1074/jbc.M210689200 12560330

[58] Rui L Yuan M Frantz D Shoelson S White MF. SOCS-1 and SOCS-3 block insulin signaling by ubiquitin-mediated degradation of IRS1 and IRS2. J Biol Chem (2002) 277(44):42394–8. doi: 10.1074/jbc.C200444200 12228220

[59] Kim J Kim C-S Jo K Lee IS Kim J-H Kim JS. POCU1b, the n-butanol soluble fraction of polygoni cuspidati rhizoma et radix, attenuates obesity, non-alcoholic fatty liver, and insulin resistance *via* inhibitions of pancreatic lipase, cAMP-dependent PDE activity, AMPK activation, and SOCS-3 suppression. Nutrients (2020) 12(12):5612. doi: 10.3390/nu12123612 PMC7759958 33255404

[60] Stuible M Tremblay ML. In control at the ER: PTP1B and the down-regulation of RTKs by dephosphorylation and endocytosis. Trends Cell Biol (2010) 20(11):672–9. doi: 10.1016/j.tcb.2010.08.013 20864346

[61] Yudushkin IA Schleifenbaum A Kinkhabwala A Neel BG Schultz C Bastiaens PIH. Live-cell imaging of enzyme-substrate interaction reveals spatial regulation of PTP1B. Science (2007) 315(5808):115–9. doi: 10.1126/science.1134966 17204654

[62] Li D Zhang S Yang C Li Q Wang S Xu X. A novel PTP1B inhibitor-phosphate of polymannuronic acid ameliorates insulin resistance by regulating IRS-1/Akt signaling. Int J Mol Sci (2021) 22(23):12693. doi: 10.3390/ijms222312693 34884501 PMC8657924

[63] Wu H Zhang T Pan F Steer CJ Li Z Chen X. MicroRNA-206 prevents hepatosteatosis and hyperglycemia by facilitating insulin signaling and impairing lipogenesis. J Hepatol (2017) 66(4):816–24. doi: 10.1016/j.jhep.2016.12.016 PMC5568011 28025059

[64] Qiu K Zheng Z Huang Y. Long intergenic noncoding RNA 00844 promotes apoptosis and represses proliferation of prostate cancer cells through upregulating GSTP1 by recruiting EBF1. J Cell Physiol (2020) 235(11):8472–85. doi: 10.1002/jcp.29690 32329523

[65] Lei K Gu X Alvarado AG Du Y Luo S Ahn EH. Discovery of a dual inhibitor of NQO1 and GSTP1 for treating glioblastoma. J Hematol Oncol (2020) 13(1):141. doi: 10.1186/s13045-020-00979-y 33087132 PMC7579906

[66] Lei X Du L Yu W Wang Y Ma N Qu B. GSTP1 as a novel target in radiation induced lung injury. J Trans Med (2021) 19(1):297. doi: 10.1186/s12967-021-02978-0 PMC8268607 34238333

[67] Wu Y Fan Y Xue B Luo L Shen J Zhang S. Human glutathione s-transferase P1-1 interacts with TRAF2 and regulates TRAF2–ASK1 signals. Oncogene (2006) 25(42):5787–800. doi: 10.1038/sj.onc.1209576 16636664

[68] Kim JH Hong Y-C. GSTM1, GSTT1, and GSTP1 polymorphisms and associations between air pollutants and markers of insulin resistance in elderly koreans. Environ Health Perspect (2012) 120(10):1378–84. doi: 10.1289/ehp.1104406 PMC3491923 22732554

[69] Dubois V Eeckhoute J Lefebvre P Staels B. Distinct but complementary contributions of PPAR isotypes to energy homeostasis. J Clin Invest (2017) 127(4):1202–14. doi: 10.1172/jci88894 PMC5373878 28368286

[70] Virtue S Petkevicius K Moreno-Navarrete JM Jenkins B Hart D Dale M. Peroxisome proliferator-activated receptor γ2 controls the rate of adipose tissue lipid storage and determines metabolic flexibility. Cell Rep (2018) 24(8):2005–2012.e2007. doi: 10.1016/j.celrep.2018.07.063 30134163 PMC6113930

[71] Hall JA Ramachandran D Roh HC DiSpirito JR Belchior T Zushin P-JH. Obesity-linked PPARg S273 phosphorylation promotes insulin resistance through growth differentiation factor 3. Cell Metab (2020) 32(4):665–675.e666. doi: 10.1016/j.cmet.2020.08.016 32941798 PMC7543662

[72] Montaigne D Butruille L Staels B. PPAR control of metabolism and cardiovascular functions. Nat Rev Cardiol (2021) 18(12):809–23. doi: 10.1038/s41569-021-00569-6 34127848

[73] Lee SM Pusec CM Norris GH De Jesus A Diaz-Ruiz A Muratalla J. Hepatocyte-specific loss of PPARg protects mice from NASH and increases the therapeutic effects of rosiglitazone in the liver. Cell Mol Gastroenterol Hepatol (2021) 11(5):1291–311. doi: 10.1016/j.jcmgh.2021.01.003 PMC8005819 33444819

