# Supplementary material for: The effect of G0S2 on insulin sensitivity: A proteomic analysis in a G0S2-overexpressed high-fat diet mouse model
Source: Front Endocrinol (Lausanne). 2023 Mar 23;14:1130350. doi: 10.3389/fendo.2023.1130350 (PMC10076770; doi:10.3389/fendo.2023.1130350)
Supplement: Supplementary file 1 [file DataSheet_1.pdf]

Supplementary Material

The Effect of *G0S2* on Insulin Sensitivity: A Proteomic Analysis in a *G0S2*-overexpressed high-fat diet mouse model

Running title: *G0S2* Treatment for Insulin Sensitivity

Dongming Wu[1]， Zhenyuan Zhang[2345]，Wenxiu Sun[6]，Yong Yan[7]，Mengzhe Jing[2345], Shizhan Ma[2345]*

*** Correspondence:** Shizhan Ma: msz2010lw@163.com

Supplementary Figures

1. Full-length blot of liver tissues protein expression level.

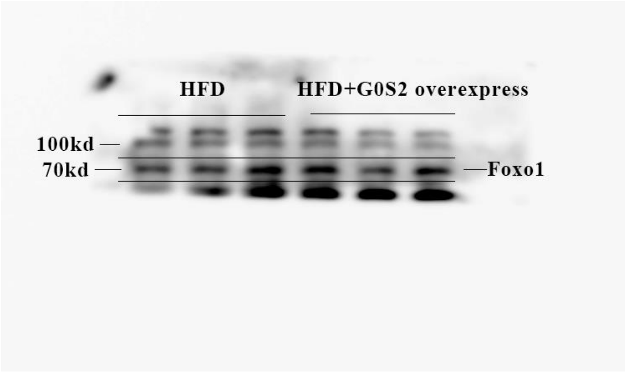

Supplementary Figure 1. Full-length blot of Foxo1 protein expression level.

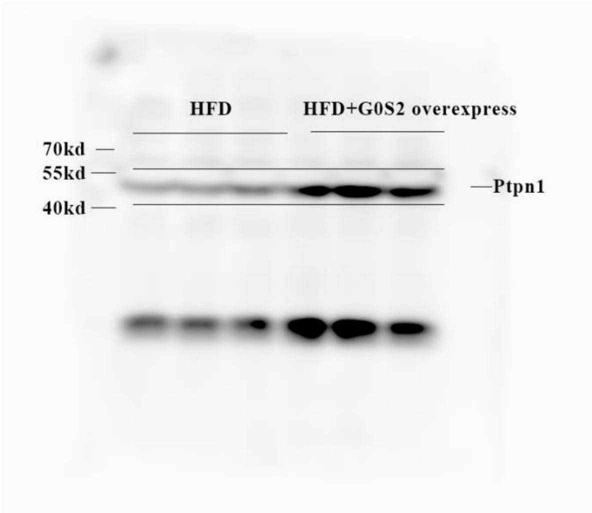

Supplementary Figure 2. Full-length blot of Ptpn1 protein expression level.

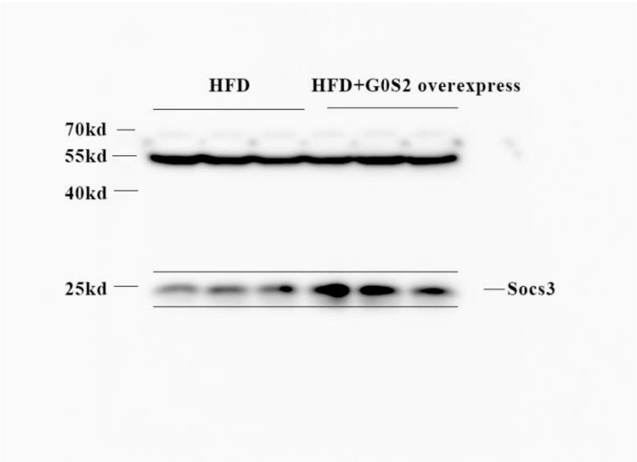

Supplementary Figure 3. Full-length blot of Socs3 protein expression level.

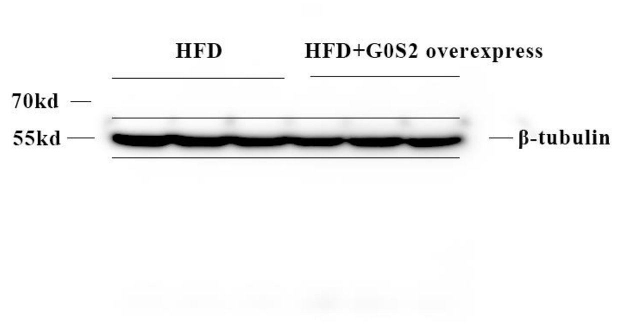

Supplementary Figure 4. Full-length blot of β-tubulin protein expression level.

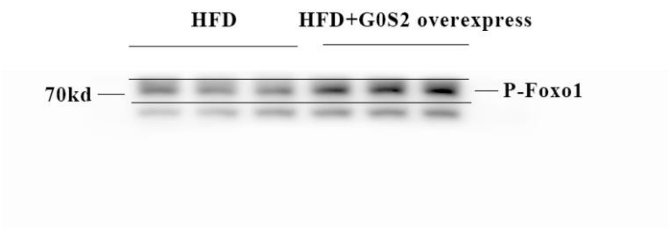

Supplementary Figure 5. Full-length blot of β-Foxo1 protein expression level.

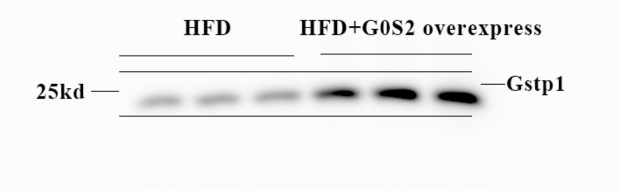

Supplementary Figure 6. Full-length blot of Gstp1 protein expression level.

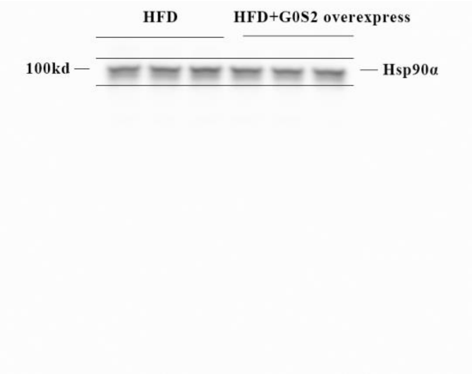

Supplementary Figure 7. Full-length blot of Hsp90 α protein expression level.

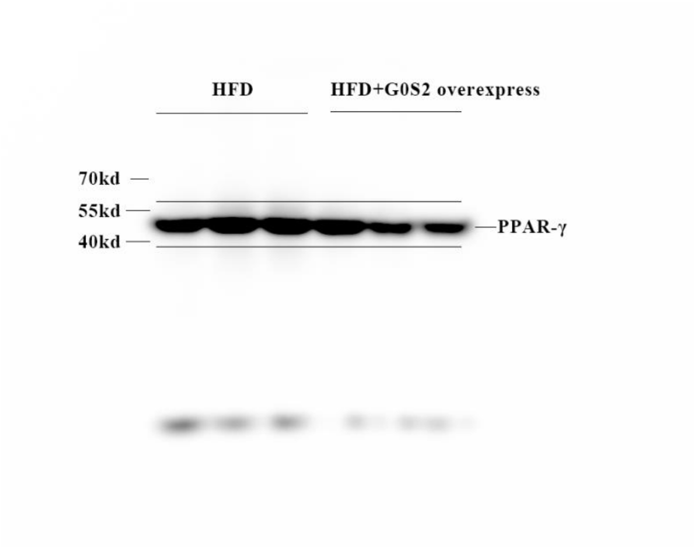

Supplementary Figure 8. Full-length blot of PPAR-γ protein expression level.

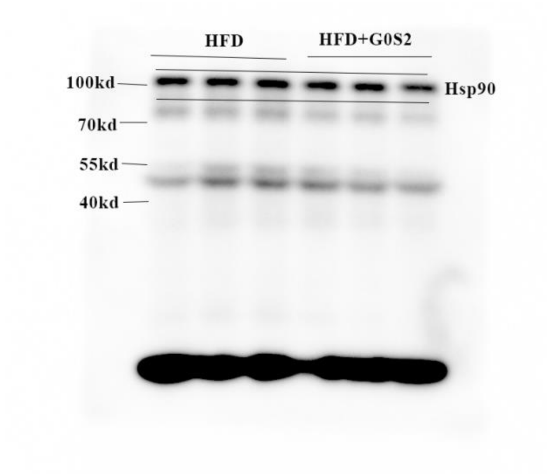

Supplementary Figure 9. Full-length blot of Hsp90 protein expression level.

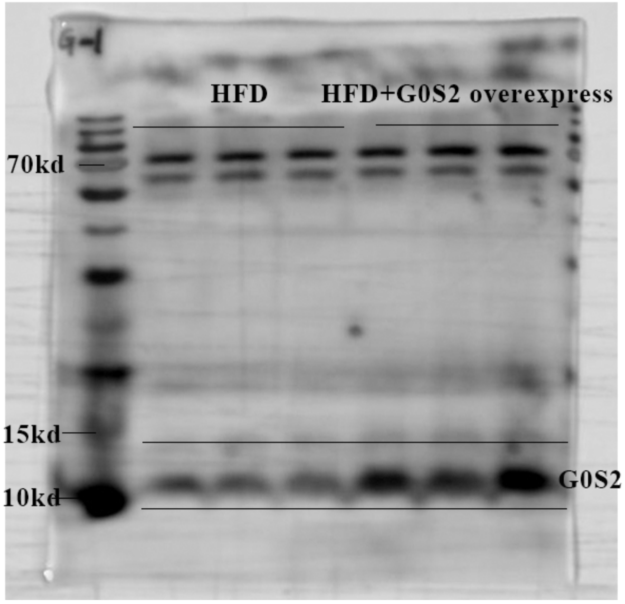

Supplementary Figure 10. Full-length blot of G0S2 protein expression level.

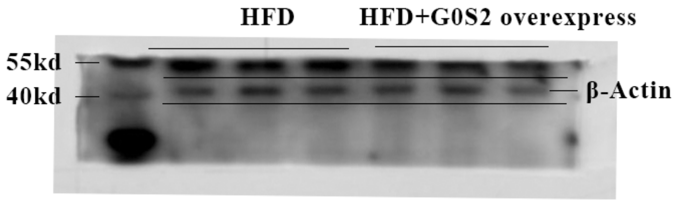

Supplementary Figure 11. Full-length blot of G0S2- β -Actin protein expression level.

2. Full-length blot of primary mouse hepatocyte protein expression level.

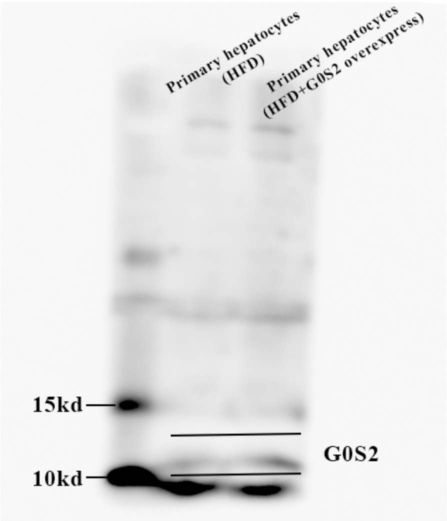

Supplementary Figure 12. Full-length blot of G0S2 protein expression level.

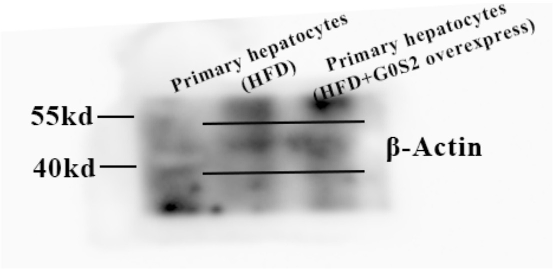

Supplementary Figure 13. Full-length blot of G0S2- β -Actin protein expression level.

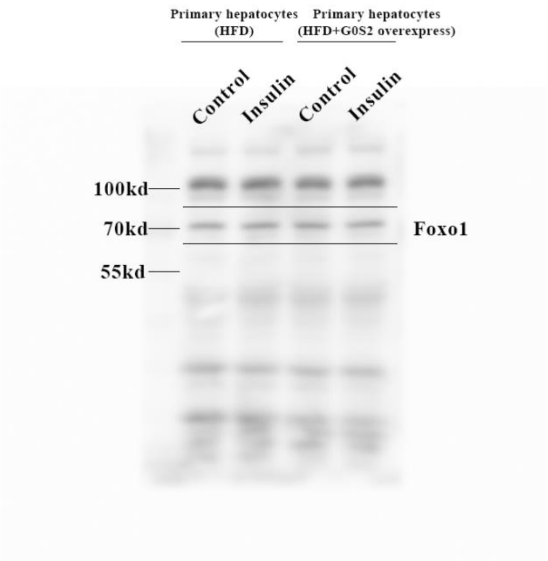

Supplementary Figure 14. Full-length blot of Foxo1 protein expression level.

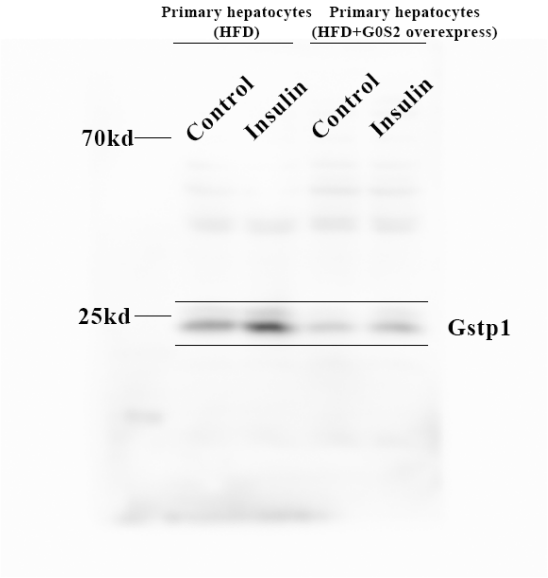

Supplementary Figure 15. Full-length blot of Gstp1 protein expression level.

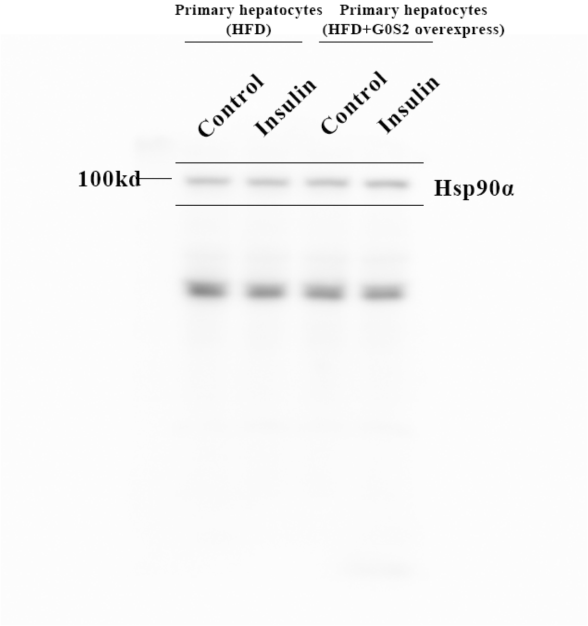

Supplementary Figure 16. Full-length blot of Hsp90α protein expression level.

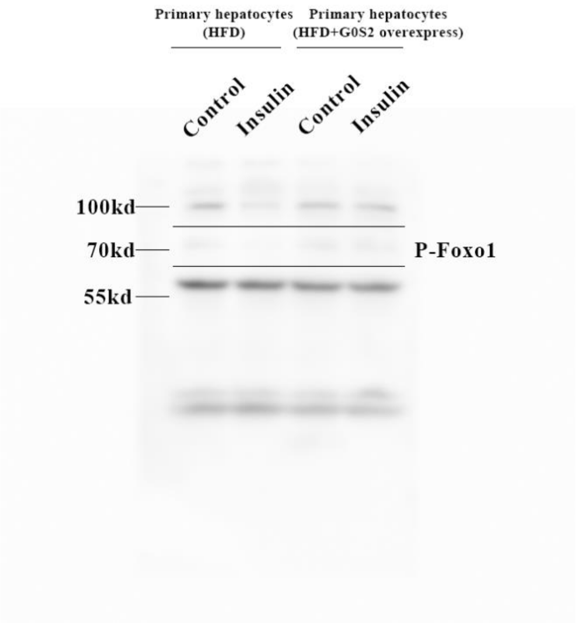

Supplementary Figure 17. Full-length blot of P-Foxo1 protein expression level.

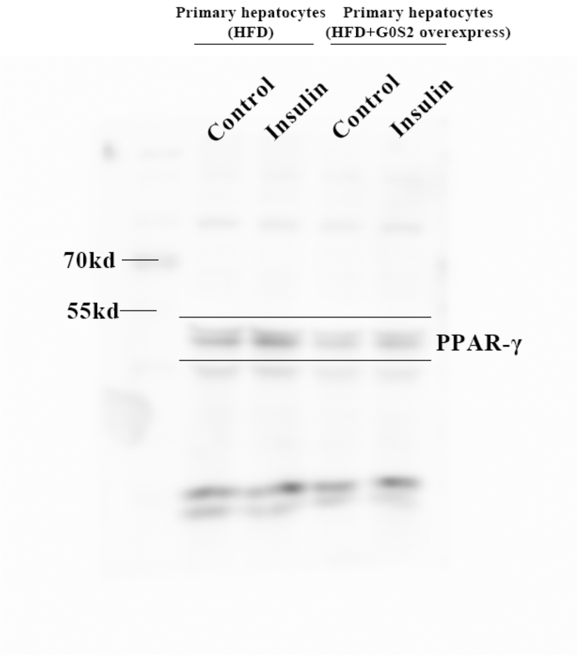

Supplementary Figure 18. Full-length blot of PPAR-γ protein expression level.

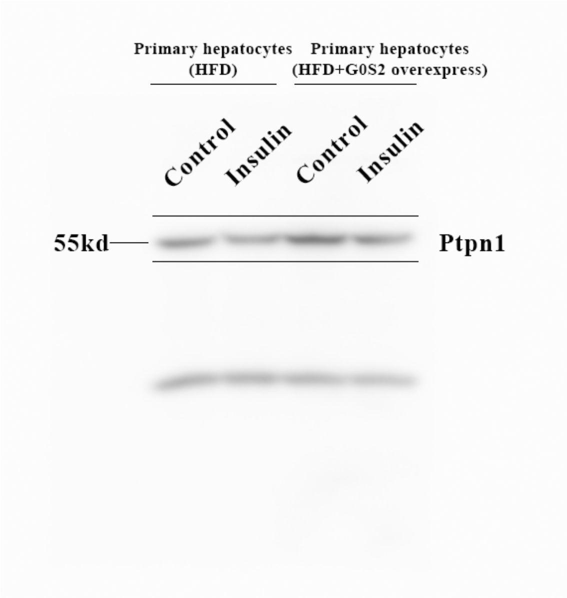

Supplementary Figure 19. Full-length blot of Ptpn1 protein expression level.

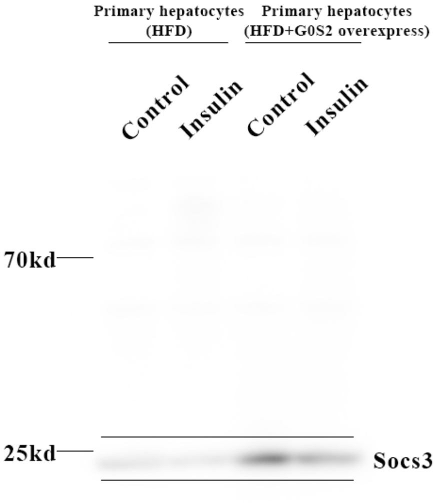

Supplementary Figure 20. Full-length blot of Socs3 protein expression level.

Supplementary table 1

The differentially expressed proteins in the liver tissue of HFD-G0S2 overexpressed mice

**Change**	**Protein IDs**	**Protein Name**	**Gene Name**	**Fold Change**
up	Q7TQI7	Ankyrin repeat and BTB/POZ domain-containing protein 2	Abtb2	62.58781577
up	Q9R1E0	Forkhead box protein O1	Foxo1	6.420271268
up	O35718	Suppressor of cytokine signaling 3	Socs3	4.792348761
up	Q9QUK6	Toll-like receptor 4	Tlr4	4.19074048
up	P11247	Myeloperoxidase	Mpo	4.102581456
up	P97393	Rho GTPase-activating protein 5	Arhgap5	4.099994191
up	Q62470	Integrin alpha-3	Itga3	3.967344651
up	Q7TQH0	Ataxin-2-like protein	Atxn2l	3.346799227
up	P27808	Alpha-1,3-mannosyl-glycoprotein 2-beta-N-acetylglucosaminyltransferase	Mgat1	3.120291894
up	P51655	Glypican-4	Gpc4	2.931553624
up	P35821	Tyrosine-protein phosphatase non-receptor type 1	Ptpn1	2.911627141
up	A2ASS6	Titin	Ttn	2.778280893

up	Q62433	Protein NDRG1	Ndrg1	2.641688724
up	Q80YD1	ATP-dependent RNA helicase SUPV3L1, mitochondrial	Supv3l1	2.61819953
up	P27601	Guanine nucleotide-binding protein subunit alpha-13	Gna13	2.586557004
up	P70335	Rho-associated protein kinase 1	Rock1	2.579219236
up	Q9JKP5	Muscleblind-like protein 1	Mbnl1	2.56523735
up	Q9CQX8	28S ribosomal protein S36, mitochondrial	Mrps36	2.408334373
up	P08101	Low affinity immunoglobulin gamma Fc region receptor II	Fcgr2	2.34607704
up	P19258	Protein Mpv17	Mpv17	2.308959022
up	P56565	Protein S100-A1	S100a1	2.173449284
up	Q920L1	Acyl-CoA (8-3)-desaturase	Fads1	2.059835232
up	Q8C863	E3 ubiquitin-protein ligase Itchy	Itch	2.030563041
up	Q9JLV5	Cullin-3	Cul3	2.000545483
up	Q8C650	Septin-10	Septin10	1.975876767
up	Q8BHI7	Elongation of very long chain fatty acids protein 5	Elovl5	1.943636596
up	Q8BGR2	Volume-regulated anion channel subunit LRRC8D	Lrrc8d	1.931676161
up	Q9CZP5	Mitochondrial chaperone BCS1	Bcs1l	1.918403268

up	Q7TMY7	Importin-8	Ipo8	1.915460475
up	Q3TC72	Fumarylacetoacetate hydrolase domain-containing protein 2A	Fahd2	1.894945607
up	Q8CAK3	Shiftless antiviral inhibitor of ribosomal frameshifting protein homolog	Shfl	1.850693054
up	O09111	NADH dehydrogenase [ubiquinone] 1 beta subcomplex subunit 11, mitochondrial	Ndufb11	1.807212622
up	Q9D0G0	28S ribosomal protein S30, mitochondrial	Mrps30	1.794549031
up	Q8BZQ7	Anaphase-promoting complex subunit 2	Anapc2	1.781631195
up	Q8CEI1	BolA-like protein 3	Bola3	1.758608045
up	Q60692	Proteasome subunit beta type-6	Psmb6	1.726763608
up	P55302	Alpha-2-macroglobulin receptor-associated protein	Lrpap1	1.721498117
up	Q8VBT2	L-serine dehydratase/L-threonine deaminase	Sds	1.718113016
up	P97434	Myosin phosphatase Rho-interacting protein	Mprip	1.664924725
up	Q61907	Phosphatidylethanolamine N-methyltransferase	Pemt	1.654503815
up	Q8K2C8	Glycerol-3-phosphate acyltransferase 4	Gpat4	1.636224246
up	Q9D1P0	39S ribosomal protein L13, mitochondrial	Mrpl13	1.626238014
up	Q8R010	Aminoacyl tRNA synthase complex-interacting multifunctional protein 2	Aimp2	1.614036145

up	P35492	Histidine ammonia-lyase	Hal	1.608594496
up	Q99KK9	Histidine--tRNA ligase, mitochondrial	Hars2	1.601407205
up	O55126	Protein NipSnap homolog 2	Nipsnap2	1.591848806
up	O08579	Emerin	Emd	1.589400753
up	Q8CIF4	Biotinidase	Btd	1.587582084
up	Q3TPX4	Exocyst complex component 5	Exoc5	1.539317868
up	P70388	DNA repair protein RAD50	Rad50	1.535942107
up	Q9JLC3	Methionine-R-sulfoxide reductase B1	Msrb1	1.531481252
up	Q9R1J0	Sterol-4-alpha-carboxylate 3-dehydrogenase, decarboxylating	Nsdhl	1.526553006
up	P40124	Adenylyl cyclase-associated protein 1	Cap1	1.519014599
up	P43006	Excitatory amino acid transporter 2	Slc1a2	1.509465859
up	P11714	Cytochrome P450 2D9	Cyp2d9	1.509100751
up	Q8BFW7	Lipoma-preferred partner homolog	Lpp	1.507287238
down	Q99KF1	Transmembrane emp24 domain-containing protein 9	Tmed9	0.658368296
down	O35343	Importin subunit alpha-3	Kpna4	0.655020207
down	Q9D883	Splicing factor U2AF 35 kDa subunit	U2af1	0.652288181

down	Q5EG47	5-AMP-activated protein kinase catalytic subunit alpha-1	Prkaa1	0.650331086
down	Q91XC9	Peroxisomal membrane protein PEX16	Pex16	0.648582282
down	O88908	Sterol O-acyltransferase 2	Soat2	0.645770033
down	Q921M7	CYFIP-related Rac1 interactor B	Cyrib	0.640751679
down	Q64331	Unconventional myosin-VI	Myo6	0.640175236
down	P61750	ADP-ribosylation factor 4	Arf4	0.636363065
down	Q11136	Xaa-Pro dipeptidase	Pepd	0.633423237
down	Q9DB43	Zinc finger protein-like 1	Zfpl1	0.630502184
down	Q99MR6	Serrate RNA effector molecule homolog	Srrt	0.6301559
down	Q9QZ85	Interferon-inducible GTPase 1	Iigp1	0.626793262
down	Q8BK75	Elongator complex protein 6	Elp6	0.622412882
down	Q923D2	Flavin reductase (NADPH)	Blvrb	0.617167042
down	O55135	Eukaryotic translation initiation factor 6	Eif6	0.616771107
down	Q9D7A8	Armadillo repeat-containing protein 1	Armc1	0.616701607
down	Q9DCM0	Persulfide dioxygenase ETHE1, mitochondrial	Ethe1	0.616061724
down	P10649	Glutathione S-transferase Mu 1	Gstm1	0.613811852
down	Q8QZY1	Eukaryotic translation initiation factor 3 subunit L	Eif3l	0.612968824

down	Q80WJ7	Protein LYRIC	Mtdh	0.605042119
down	O88874	Cyclin-K	Ccnk	0.598541678
down	Q9CX34	Protein SGT1 homolog	Sugt1	0.593365001
down	Q9CQI6	Coactosin-like protein	Cotl1	0.589188456
down	P28076	Proteasome subunit beta type-9	Psmb9	0.584247003
down	Q80W54	CAAX prenyl protease 1 homolog	Zmpste24	0.5795569
down	Q9CXY6	Interleukin enhancer-binding factor 2	Ilf2	0.579548446
down	Q07797	Galectin-3-binding protein	Lgals3bp	0.5793389
down	Q8CIN4	Serine/threonine-protein kinase PAK 2	Pak2	0.578505093
down	Q3UH53	Protein sidekick-1	Sdk1	0.576685199
down	Q9QWR8	Alpha-N-acetylgalactosaminidase	Naga	0.574780265
down	Q9ER73	Elongator complex protein 4	Elp4	0.571214136
down	P19157	Glutathione S-transferase P 1	Gstp1	0.563400004
down	P57716	Nicastrin	Ncstn	0.562055528
down	P60603	Reactive oxygen species modulator 1	Romo1	0.559714141
down	Q99LC9	Peroxisome assembly factor 2	Pex6	0.556661912
down	Q5KU39	Vacuolar protein sorting-associated protein 41 homolog	Vps41	0.5554666

down	Q8VC49	Interferon alpha-inducible protein 27-like protein 2B	Ifi27l2b	0.542415019
down	Q60631	Growth factor receptor-bound protein 2	Grb2	0.526383608
down	Q9JI39	ATP-binding cassette sub-family B member 10, mitochondrial	Abcb10	0.520913684
down	Q8BFY6	Peflin	Pef1	0.498583001
down	Q91WL5	Cytochrome P450 4A12A	Cyp4a12a	0.49784561
down	Q8R5H1	Ubiquitin carboxyl-terminal hydrolase 15	Usp15	0.492130859
down	Q9CR24	Nucleoside diphosphate-linked moiety X motif 8	Nudt8	0.491745608
down	P62821	Ras-related protein Rab-1A	Rab1A	0.456201888
down	Q9DBW0	Cytochrome P450 4V2	Cyp4v2	0.449213586
down	Q9CQM5	Thioredoxin domain-containing protein 17	Txndc17	0.434000159
down	Q9D154	Leukocyte elastase inhibitor A	Serpinb1a	0.430447983
down	P00493	Hypoxanthine-guanine phosphoribosyltransferase	Hprt1	0.415656889
down	Q9D4G2	Heat shock factor 2-binding protein	Hsf2bp	0.406455141
down	Q9WVT6	Carbonic anhydrase 14	Ca14	0.399987949
down	P37238	Peroxisome proliferator-activated receptor gamma	Pparg	0.397358829
down	E9Q3L2	Phosphatidylinositol 4-kinase alpha	Pi4ka	0.375182016

down	Q9CXS4	Centromere protein V	Cenpv	0.350370626
down	P84096	Rho-related GTP-binding protein RhoG	Rhog	0.328422711
down	Q64459	Cytochrome P450 3A11	Cyp3a11	0.31724434
down	Q8VI94	2-5-oligoadenylate synthase-like protein 1	Oasl1	0.316828319
down	O70343	Peroxisome proliferator-activated receptor gamma coactivator 1-alpha	Ppargc1a	0.315919544
down	Q91ZS8	Double-stranded RNA-specific editase 1	Adarb1	0.315379096
down	Q9DBE0	Cysteine sulfinic acid decarboxylase	Csad	0.315010582
down	Q9Z0N2	Eukaryotic translation initiation factor 2 subunit 3, Y-linked	Eif2s3y	0.296742219
down	Q3U182	CREB-regulated transcription coactivator 2	Crtc2	0.275179445
down	Q9D358	Low molecular weight phosphotyrosine protein phosphatase	Acp1	0.273314716
down	Q78IQ7	Zinc transporter ZIP4	Slc39a4	0.247186647
down	Q8BUE4	Ferroptosis suppressor protein 1	Aifm2	0.238349496
down	Q9D1M4	Eukaryotic translation elongation factor 1 epsilon-1	Eef1e1	0.223184477
down	Q3U3V8	X-ray radiation resistance-associated protein 1	Xrra1	0.103342257
down	Q8K2F8	Protein LSM14 homolog A	Lsm14a	0.097406908

down	Q5RL79	Keratinocyte-associated protein 2	Krtcap2	0.043668499

Up, upregulated; down, downregulated.

Supplementary table 2 Primer sequences for qPCR

Gene	Sequence (5'-3')	
Mouse *G0S2*	F:	GTGAAGCTATACGTGCTGGG
	R:	CCGTCTCAACTAGGCCGAG
Mouse *GAPDH*	F:	GTAGTGATGGTATGGATTGTGGTTT
	R:	CTCAAAATTATCAACAATACATCCTACA
Mouse *Ptpn1*	F:	CGGCTATTTACCAGGACATTC
	R:	TGCGGTTGAGCATGACCAC
Mouse *Socs3*	F:	CTCCCCGTTGTGAAGAGGCA
	R:	CTGGGCTCCAAGATGGCTCA
Mouse *Foxo1*	F:	CAAAGTACACATACGGCCAATCC
	R:	CGTAACTTGATTTGCTGTCCTGAA
Mouse *Gstp1*	F:	TTTAGTATTGTGGTCGATTCGG
	R:	TATAAAAAACATTACGCTCCGC
Mouse *Pparγ*	F:	TCTGCAGATCGCGTGGAG
	R:	CTTGTCCCGGCATAGCAAC
Mouse *β-actin*	F:	ATGGATGACGATATCGCTG
	R:	GTTGGTAACAATGCCATGTTC-
